# Predicted Effects of Climate Change on Future Distributions of Ectomycorrhizal Fungi

**DOI:** 10.1002/ece3.72743

**Published:** 2025-12-17

**Authors:** Muyao Qi, Martin I. Bidartondo, Laura M. Suz, C. David L. Orme, Ricardo Arraiano‐Castilho, Carolina Tovar

**Affiliations:** ^1^ Imperial College London London UK; ^2^ Royal Botanic Gardens, Kew Richmond UK; ^3^ Department of Ecology and Evolution University of Lausanne Lausanne Switzerland

**Keywords:** climate change, distribution change, ectomycorrhizal fungi, environmental factors, forests, host specificity, species distribution models

## Abstract

To model the distribution of ectomycorrhizal (ECM) fungi to (i) analyse climate change impacts on their future distribution areas and centroids and (ii) analyse their distribution changes by ECM fungal host specificity. Location: Europe. Time period: 2041–2100. Major taxa studied: Ectomycorrhizal fungi. We modelled the distributions of 60 common ECM fungal species in European forests and projected their future distributions under three different shared socioeconomic pathways (SSP126, SSP370 and SSP585) for 2041–2070 and 2071–2100. Both abiotic and biotic (host tree distribution) variables were included in the modelling, with ECM fungal species classified into broadleaf specialists (19), conifer specialists (22) and generalists (19). We estimated changes in both the areas and geographic centroids between the projected future and current distributions for each species and for each ECM fungal host specificity group. We found that host tree distributions make strong contributions to ECM fungal distribution models, but their influence varied with ECM fungal host specificity. The distributions of most ECM fungal species are projected to decline (ranging from 0.2% to 64%) and shift northward under the three climate scenarios in both 2041–2070 and 2071–2100, and most ECM fungal conifer specialists are projected to lose more of their current distribution compared to broadleaf specialists and generalists. Substantial decline of studied ECM fungal co‐occurrence is projected in southern England, central Europe, Finland and Sweden. Our results evidence ECM fungi will be mostly negatively affected by climate change, but this will vary with host specificity. Thus, conservation actions need tailored actions for the different groups. Conifer specialists need special attention, either through targeted monitoring or by assessing their conservation status. Overall, a conservation plan for fungi is needed under climate change scenarios.

## Graphical abstract

This study modelled the future distributions of 60 common ECM fungi in Europe and explored the relationship between ECM fungal distribution changes and host specificity. The results reflect that the distributions of most fungi will decrease and shift under climate change, and more conifer specialists will lose their current habitats compared to broadleaf specialists and host generalists.

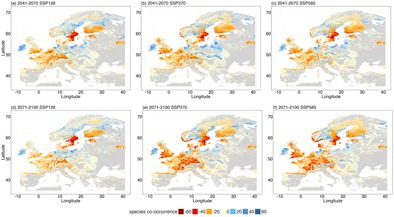

## 1 Introduction

Ectomycorrhizal (ECM) fungi are essential in temperate, boreal and some tropical forests (Tedersoo et al. 2010; Peay and Matheny 2016). They form symbioses with plant roots, providing nitrogen (N), phosphorus (P) and water, in return for plant carbohydrates, thus playing a crucial role in global carbon (C) and nutrient cycling (Smith and Read 2008; Tedersoo et al. 2014; van der Heijden et al. 2015; Peay and Matheny 2016). Ectomycorrhizal fungi can also benefit host plants by improving their tolerance to drought and soil salinity and by protecting host plants against pathogens and osmotic stress (Branzanti et al. 1999; Smith and Read 2008; Guerrero‐Galán et al. 2019; Aryal et al. 2021; Wang et al. 2021). In addition, the mycelial networks that ECM fungi form in the soil can promote the survival of colonised plant seedlings (Teste et al. 2009). Despite the importance of ECM fungi, the present and future distributions of most ECM fungi remain little explored. A few studies have focused on modelling the distributions of one ECM fungus or a group of species but at a small scale (Wollan et al. 2008; Guo et al. 2017), or usually using only abiotic variables to model fungal distributions (Pietras et al. 2018), with less consideration of the influence of biotic variables on these models.

Ectomycorrhizal fungal distributions can be influenced not only by abiotic environmental conditions but also critically by the availability of their host plants. Temperature, precipitation, soil N and pH have been shown to influence ECM fungal distributions (Wollan et al. 2008; Guo et al. 2017; Větrovský et al. 2019; Qi et al. 2024). Although less studied, a few studies have found that the dominant host tree species, host tree age and human disturbance could also play an important role in determining the distribution of ECM fungal fruitbodies such as mushrooms (Yu et al. 2021). However, there is still a knowledge gap regarding the role of host trees in shaping ECM fungal species distributions at continental scales (Bidartondo et al. 2018). In addition, ongoing climate change and future projections suggest an increase in the frequency of extreme events, such as heatwaves, droughts and heavy precipitation (IPCC 2023). How these changes will impact future ECM fungal distributions, and therefore their functions, also remains unknown.

Modelling species distributions has become a key tool in conservation of endangered, endemic, native and economically important species (Bazzichetto et al. 2018; Thapa et al. 2018; Lozano‐Jaramillo et al. 2019; Stevens and Conway 2020) by combining species locations and related environmental data through modelling algorithms (Guisan et al. 2017). Nevertheless, a single algorithm may not have the same performance across different species (Segurado and Araújo 2004), and its model performance could be lower than an ensemble model (Grenouillet et al. 2011), which combines several individual algorithms (Araújo and New 2006). Thus, ensemble models have become popular tools for modelling the distributions of multiple species simultaneously and independently (Grenouillet et al. 2011). While species distribution models have been commonly used to model the distribution of animals and plants, their use to project ECM fungal distributions in Europe under climate change scenarios is uncommon. This is primarily due to data quality and availability; publicly available occurrence data are strongly biased towards overrepresentation of species with edible or large above‐ground fruitbodies and lack direct data on below‐ground fungus‐root associations. However, a recent study suggests combining the fruitbody data which is commonly recorded in databased collecting occurrences (e.g., GBIF) and root data (e.g., DNA sequencing data from ectomycorrhizas) of each ECM fungus to improve the accuracy of distribution modelling (Qi et al. 2024).

Understanding the impact of climate change on European ECM fungal distributions could inform conservation decisions for threatened fungal species and forest management and help predict the influences of climate change on tree mineral nutrition, as ECM fungi affect latter (Suz et al. 2021). In this study, we selected 66 commonly detected ECM fungal species from a European below‐ground ECM root data set (van der Linde et al. 2018) to model their distributions and to project their distributions under different future climate change scenarios. These ECM fungal species were classified based on their host specificity as host specialists (ECM fungal species that colonised only the roots of broadleaf (e.g., European beech, pedunculate and/or sessile oak) or conifer trees (Norway spruce and/or Scots pine)), and host generalists (those which were found colonising both broadleaf and conifer tree roots [at least one broadleaf tree and one conifer tree]). As the current habitats may become less favourable for many ECM fungi under increased temperature and the distribution of host trees may decline under climate change (Dyderski et al. 2017; Guo et al. 2017; van der Linde et al. 2018; Buras and Menzel 2019), we hypothesised that:

1. The projected ECM fungal future distributions will decrease under climate change, with a stronger reduction for host specialists than host generalists (H1).
2. The projected future distributions of ECM fungi will shift north under climate change as measured by their geographic centroid, especially for host specialists (H2).

## 2 Methods

### 2.1 Ectomycorrhizal Fungal Species

Sixty‐six ECM fungal species (27 genera, 18 families, two phyla) were selected based on the ECM dataset (full ITS rDNA sequences) from van der Linde et al. (2018). This dataset was derived from the below‐ground sampling of ectomycorrhizas across 136 Level II plots belonging to the International Cooperative Programme on Assessment and Monitoring of Air Pollution Effects on Forests (ICP Forests, http://icp‐forests.net) in 20 European countries and each species occurred in at least five plots. Each ECM fungal species in this ECM dataset corresponds to a single operational taxonomic unit (OTU) identified against the UNITE v7 database (Kõljalg et al. 2013; Nilsson et al. 2019) with a unique species hypothesis number and species name as in the original study. Information on the species hypothesis numbers and their corresponding names is available in our previous study (Qi et al. 2024). We used the names of the selected 66 ECM fungal species to download their fruitbody occurrence records from public databases. In the ECM dataset, five host trees were recorded from the ECM fungal plots, including three broadleaf trees—European beech (*Fagus sylvatica*), pedunculate and sessile oak ( *Quercus robur* and *Q. petraea* ) and two conifer trees—Norway spruce ( *Picea abies* ) and Scots pine ( *Pinus sylvestris* ). We classified the 66 ECM fungal species into broadleaf specialists (19), conifer specialists (25) and host generalists (22) based on their recorded host trees in the ECM dataset. In these 66 species, two only colonised European beech, four only oak and four only Scots pine, while the 22 host generalists colonised all five tree species.

### 2.2 Occurrence Data of ECM Fungi

We combined fruitbody and root occurrence data for each fungal species to better represent their ecological niche, following our previous study (Qi et al. 2024). For the fruitbody data set, the geographical coordinates of observations for the 66 fungal species were downloaded from the Global Biodiversity Information Facility (GBIF; GBIF.org 2021) and PlutoF API (Abarenkov et al. 2010) for the UNITE database, both of which comprise human observations and preserved specimens, totalling 569,973 records. For the root dataset, the geographical coordinates of the site where the ECM fungal species were detected were selected from van der Linde et al. (2018). To minimise spatial sampling bias, we reduced the combined species data to single observations per environmental variable pixel (2.5 arc minutes). The filtered whole ECM fungal occurrence data that were used in this study was 160,735.

### 2.3 ECM Model Predictors

#### 2.3.1 Abiotic Predictors

Six abiotic predictors were selected for modelling ECM fungal distributions as they have been identified as key factors influencing ECM fungal communities (Suz et al. 2014; van der Linde et al. 2018; Vasco‐Palacios et al. 2020), which had positive and significant correlation with locally collected climate data in ICP Forests plots from van der Linde et al. (2018) and Qi et al. (2024). These abiotic environmental variables included three climate variables downloaded from CHELSA (30 arcsec, ~1 km spatial resolution; Karger et al. 2017; https://chelsa‐climate.org) and three soil variables downloaded from the World Soil Information database (at 250 m spatial resolution with mapped units, ISRIC, https://www.isric.org). The variables were mean annual air temperature (bio1), isothermality (bio3), annual precipitation amount (bio12), soil total nitrogen (sN), soil pH (ph) and soil organic carbon stock (ocs) (soil depth layer: 5–15 cm). Future climate variables derived from the GFDL‐ESM4 model, which is the best ranked in CHELSA, were downloaded for the periods 2041–2070 and 2071–2100 under three shared socioeconomic pathways (SSP126, SSP370 and SSP585). All abiotic variables were cropped to the extent of Western Europe (W‐11, E41, S35, N72) and resampled to 2.5 arcmin resolution in WGS84 projection using the ‘*raster*’ R package (Hijmans et al. 2023).

#### 2.3.2 Biotic Predictors

We also included the ECM fungal host tree distribution as a predictor. Each tree species distribution was modelled, and future distributions were projected under the same future climate scenarios used for the fungal data set. The predictors of host tree distribution models were: mean diurnal air temperature range (bio2), temperature seasonality (bio4), mean daily mean air temperatures of the wettest quarter (bio8), precipitation seasonality (bio15), mean monthly precipitation amount of the warmest quarter (bio18), mean monthly precipitation amount of the coldest quarter (bio19), soil total nitrogen (sN) and pH (soil depth layer: 5–15 cm). We used four algorithms, including generalised linear model (GLM), generalised boosting model (GBM), random forest (RF) and maximum entropy (Maxent), to build an ensemble model. Each algorithm was trained with 10,000 randomly generated pseudo‐absence points and ran with five‐fold cross validation for each species. Generated individual models from each algorithm were evaluated by true statistic skill (TSS) and relative operating characteristic (ROC), and each produced ensemble model was evaluated by TSS. The binary (presence/absence) maps, which we used as predictor layers in the ECM fungal models, were derived using the maximum TSS threshold. Details of host tree distribution modelling are provided in Data S1 (i.e., Host tree distribution modelling).

### 2.4 ECM Fungal Species Distribution Models

We tested the contribution of host tree distributions in ECM fungal distribution models by using two types of models. One model only used the abiotic predictors, while the other used the abiotic predictors and the fungal host tree distributions. For each species, we built an ensemble model using four algorithms: GLM, GBM, RF and Maxent. Each algorithm was trained with 10,000 randomly generated pseudo‐absence points in the study area with the ‘*biomod2* (*v4.1–2*)’ R package (Barbet‐Massin et al. 2012; Thuiller et al. 2023; details of parameter setting in Table S4). The generated model from each algorithm was fitted with five‐fold cross validation, training on 80% of the data and testing on 20% in each fold. The model performance was then evaluated by TSS and ROC. The TSS ranges from −1 to 1, with a value close to 1 indicating better performance of the model (Allouche et al. 2006). The ROC values range from 0 to 1, where 0.5 indicates the model is not better than a random one and 1 indicates perfect performance (Zhang et al. 2015). We retained individual models with TSS values above 0.7 and ROC values above 0.8 for building an ensemble model. Each produced ensemble model was evaluated by TSS, and only those with high evaluation scores (TSS ≥ 0.7) were used in subsequent analyses (hereafter referred to as valid ensemble models; Table S5). The future distribution of each species was projected under three different shared socioeconomic pathways (SSP126, SSP370 and SSP585) for both 2041–2070 and 2071–2100 by using the future climate variables, projected future host tree distributions under the same time period and climate scenario, and assuming static soil variables. We converted continuous maps to binary ones by using the maximum TSS threshold.

### 2.5 Analysis of Distribution Changes

For species with valid ensemble models (i.e., where TSS ≥ 0.7), we calculated the geographic centroids of their current and future distributions using the ‘*rgeos*’ R package (Bivand et al. 2017). We then calculated the centroid shift distance and direction (angles) between current and each future distribution per species, and per grouping results by host specificity of ECM fungi. For visualising these shifts, we set the current distribution centroids as the origin of the coordinate axes (0, 0) and coloured the future distribution centroids under each climate scenario. The distance between the axes (0, 0) and each future distribution centroid represents the shift distance of distribution centroids for each species.

We calculated the relative changes in distribution area for each species between the future and present (baseline) using the equation: (Area_future_—Area_baseline_)/Area_baseline_ × 100% for each of the six future distributions. Negative values indicate range contraction, with −1 representing complete loss. The average change in distribution area for those ECM fungal species was calculated based on host specificity. We mapped changes in species co‐occurrences by subtracting the summed current species distributions from the summed future distributions of each pixel. The resulting values ranged from (−60, 60), where −60 indicates loss of all 60 species in a pixel under that climate scenario. We also calculated species co‐occurrence by using the continuous probability distribution maps (ranging from −60,000 to 60,000). All numerical values were rounded to one decimal place for consistency.

## 3 Results

### 3.1 Tree Distribution Models

We modelled the distribution of five known host trees of ECM fungi and projected their present and future distributions. Under future climate change, most host tree distribution areas were projected to be reduced and their distribution centroids were projected to shift northward for all periods and scenarios, except for *F. sylvatica* and *Q. petraea* , which distribution areas were projected to increase under all climate scenarios in 2041–2070, and *Q. robur* , which shifted southwest (Figure S4; Table S9).

### 3.2 Comparison Between Models Using Only Abiotic Predictors and Models Including Host Distributions

**FIGURE 1:**
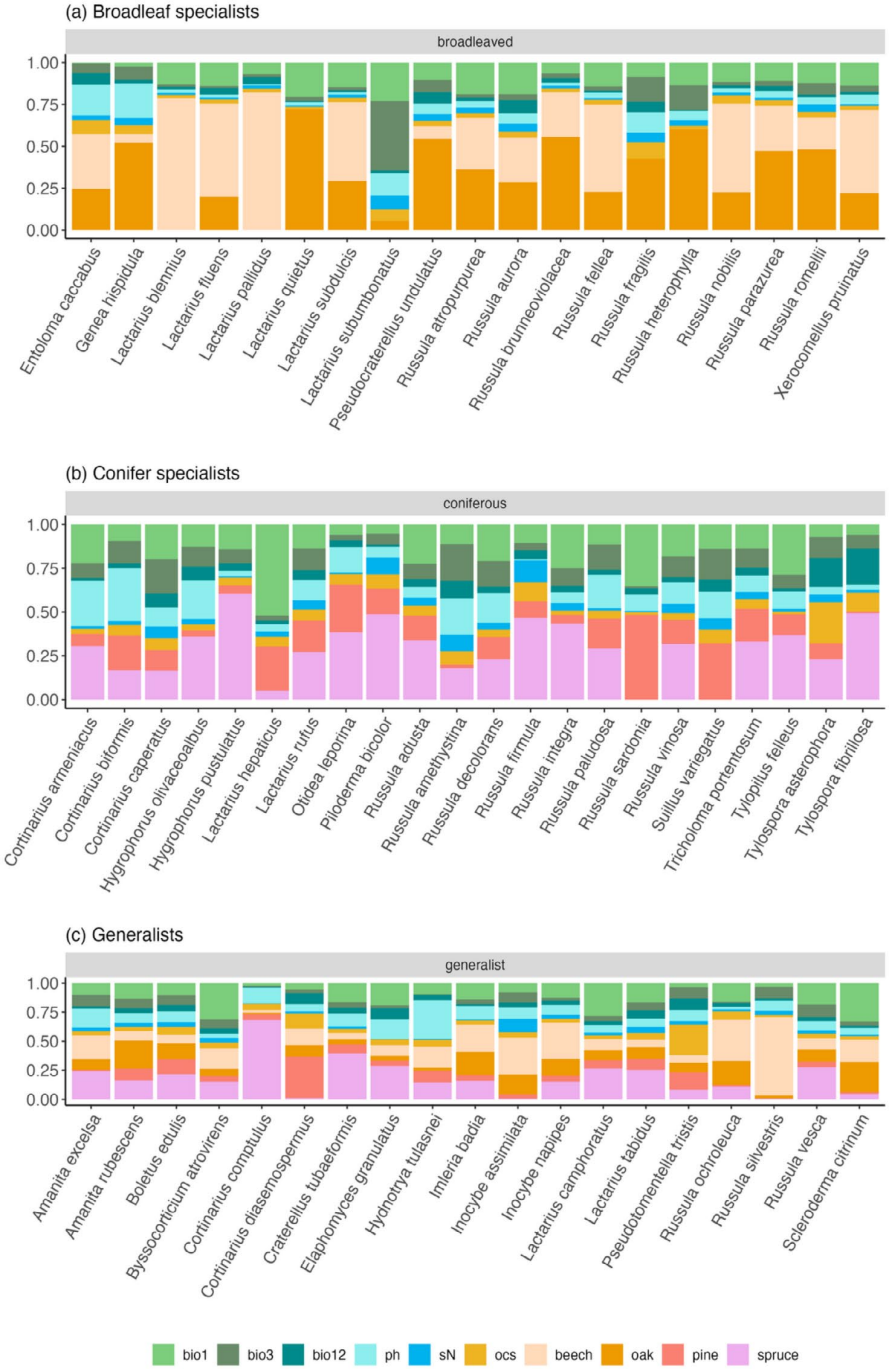
Relative importance of each variable on the distribution models of 60 ectomycorrhizal fungal species with valid ensemble models. beech, distribution of European beech; bio1, mean annual air temperature; bio12, annual precipitation amount; bio3, isothermality; oak, distributions of pedunculate and sessile oak; ocs, soil organic carbon stock; ph, soil pH; pine, distribution of Scots pine; sN, soil total nitrogen; spruce, distribution of Norway spruce.

Our results showed that models which used host tree distributions as predictors performed significantly better than models which only used abiotic predictors (Figure S1). These models showed that host tree distributions had a strong influence on shaping ECM fungal distributions (Figure 1; Figures S1–S3; Tables S1–S3). Therefore, the rest of the results we present here are based on these models. Details of the comparison between these two models are provided in Data S1 (e.g., Comparison of ECM fungal distribution models).

The models that included host tree distributions as a predictor were run for 66 ECM fungal species; however, models failed to produce valid ensemble models for six species (TSS = NA or TSS < 0.7; Table S5). This resulted in having valid models for 60 species (19 broadleaf specialists, 22 conifer specialists and 19 generalists) for the subsequent analyses.

### 3.3 Future Projected ECM Fungal Distribution Changes

By 2041–2070, most ECM fungal species are projected to lose distribution area: 57 under SSP126, 50 under SSP370 and 54 under SSP585, respectively (Table S6). Among these species, *Cortinarius armeniacus* and *Russula vinosa* are projected to lose more than half of their current distribution area under SSP370 or SSP585. Broadleaf specialists showed the smallest average decline (−7.7%, −7.2%, −11.4% under SSP126, SSP370, SSP585, respectively), followed by generalists (−17%, −18.9%, −20.3%) and conifer specialists (−20%, −32.9%, −32.9%) (Table 1).

**TABLE 1:** Average change in distribution area for 60 ectomycorrhizal fungal species under future climate change scenarios based on host specificity.

Time period	Scenario	Broadleaf specialists (%)	Conifer specialists (%)	Generalists (%)
2041–2070	SSP126	−7.7	−20.0	−17.0
	SSP370	−7.2	−32.9	−18.9
	SSP585	−11.4	−32.9	−20.3
2071–2100	SSP126	−10.1	−19.5	−18.9
	SSP370	−31.6	−42.7	−36.2
	SSP585	−30.3	−42.5	−35.9

By 2071–2100, distribution losses are predicted to intensify: 58 species decrease their distribution area under SSP126, 58 under SSP370 and 57 under SSP585 (Table S6). It is worth noting that 11 ECM host specialists are projected to lose more than half of their current distribution area under SSP370 and SSP585 (Table S6). The average decrease rates of all ECM fungi are higher than those in 2041–2070 under the same climate scenario (Table 1). Similarly, broadleaf specialists' distribution areas are least affected, while conifer specialists' contract the most (Table 1).

Notably, the generalist *Cortinarius comptulus* is projected to lose most of its distribution area under SSP126 (31.9% in 2041–2070 and 33.0% in 2071–2100) (Table S6). Under SSP370, the conifer specialist *R. vinosa* and broadleaf specialist *Xerocomellus pruinatus* are projected to lose 50.4% and 60.8% of their current distribution area, respectively, which is more than any of the other species under SSP370 in 2041–2070 and 2071–2100 (Table S6). The conifer specialists, *R. vinosa* and *C. armeniacus* are the species that would lose the most distribution area among all species under SSP585 in 2041–2070 and 2071–2100, 53.1% and 64.4%, respectively (Table S6). *Lactarius subumbonatus* is the only species projected to increase its range under all climate scenarios and time periods (Table S6). On the other hand, not all species show a monotonic response in their distribution changes under climate change scenarios. For example, *Suillus variegatus* is projected to show an expansion of 0.2% under SSP126 followed by subsequent contractions of 13.0% and 11.3% under SSP 370 and SSP585, respectively, in 2041–2070 (Table S6).

In addition to the changes in the distribution area, the distribution centroids of most species are projected to shift southwest or northeast under all scenarios. The centroids of most conifer specialists move further northeast (Figure 1b; Tables S7 and S8), while some broadleaf specialists shift further southwest, and generalists shift northeast and southwest evenly under the three climate scenarios (Figure 1a,c; Tables S7 and S8).

### 3.4 Changes in Species Co‐Occurrences

At present, most of the 60 species co‐occur in central Europe and western Sweden (Figure S5). In 2041–2070, most of Europe is projected to lose 20 to 40 of our studied species, while northern Europe may gain fewer than 20 under climate change (Figure 3). The losses of species are projected to occur mainly in southern England, central Europe, Finland and southeastern Sweden under SSP126 (Figure S6a); while gains in area may occur in Ireland, northern Germany, Poland, Sweden and Finland (Figure 3a). Higher CO_2_ emissions (SSP370 and SSP585) exacerbate losses in these regions (Figure 3; Figure S6b,c).

By 2071–2100, the loss of species is projected to intensify in central Europe, Sweden and the UK, especially under SSP 370 and SSP 585 (Figure 3d–f; Figure S6d–f). The southeastern coast of Sweden is projected to lose nearly all the studied species across all climate scenarios (Figure 3d–f; Figure S6d–f). Species co‐occurrence based on the probability distributions of ECM fungi did not differ importantly from those calculated based on the binary distributions under each climate scenario (Figure S7).

## 4 Discussion

**FIGURE 2:**
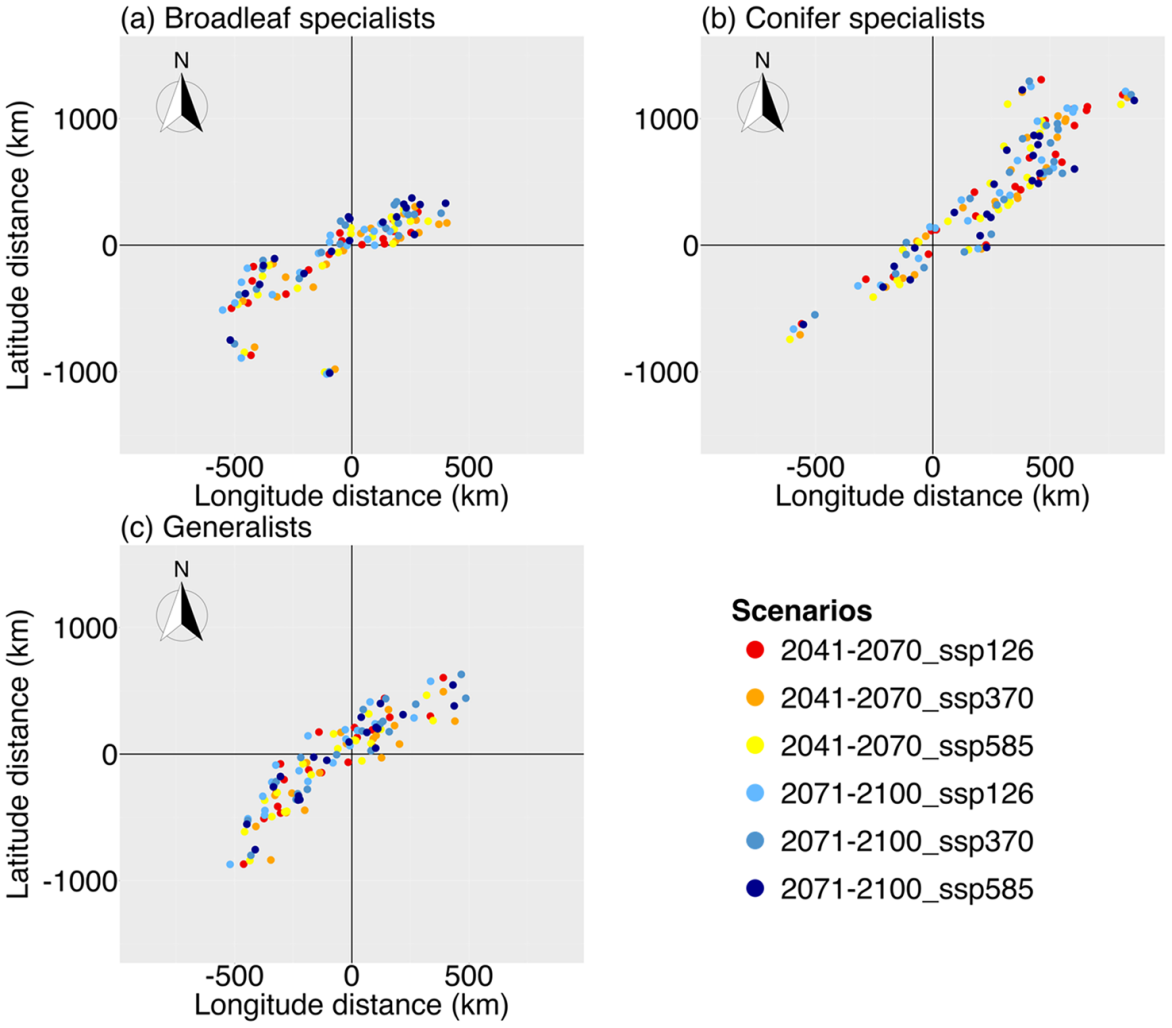
Shifts in distribution centroids of ectomycorrhizal fungi under three SSPs for 2041–2070 and 2071–2100. (a), (b) and (c) show the shifts of distribution centroids for broadleaf specialists, conifer specialists and generalists, respectively. The point (0, 0) represents current distribution centroids, and the coloured points represent future distribution centroids. The distance between (0, 0) and each coloured point represents the distance when comparing present day centroid and future projected ones for each species.

This study shows that incorporating host tree distribution improves the performance of ECM fungal distribution models and highlights the critical role of host trees in shaping fungal distributions, especially for host specialists (Figures S1– S3; Tables S1–S3). Our analysis indicates that the future distribution area of most studied ECM fungi is projected to decline under climate change scenarios (Table 1; Table S6). Distribution centroids generally shift northwards, mostly for conifer specialists (Figure 2), and central Europe is projected to lose most of the studied species under all climate scenarios (Figure S6). These results largely support our H1 and H2 that ECM fungi are projected to have a decline in distribution areas and their distribution centroids are projected to move northward under climate change.

### 4.1 Future Distributions of ECM Fungi

Most of the studied 60 ECM fungi are projected to lose distribution area, as we hypothesised (H1), though a few expand (Table S6). For example, *X. pruinatus* (broadleaf specialist) is projected to lose over 60% of its current distribution under scenarios SSP370 and SSP585 by 2071–2100 (Table S6). While not highly valued for food, this species contributes to ECM fungal diversity and serves as a positive bioindicator of tree nutrition (van der Linde et al. 2018). Its projected distribution decline may therefore signal reduced ECM fungal diversity and potential disruptions in P acquisition under elevated N deposition and climate change. Economically and culturally important fungi such as the generalist *Boletus edulis* are also vulnerable, with projected losses of about 46% under SSP370 and SSP585 by 2071–2100. A decline in prized edible fungi like *B. edulis* may directly affect rural livelihoods, commercial harvests and foraging traditions, while also reducing ecological services linked to nutrient or C cycling and forest productivity. These results highlight that losses in ECM fungi represent both ecological risks and societal challenges, underscoring the need to integrate fungi into forest management and conservation planning. From an ecological perspective, declines in ECM fungal distributions may reduce the capacity of trees to establish in new areas, especially if key symbionts are absent or slow to disperse.

By contrast, *L. subumbonatus* (broadleaf specialist) is the only species projected to increase its range under all climate scenarios and time periods (Table S6). This may reflect relatively stable host availability combined with tolerance to broader climatic or edaphic conditions. Such patterns emphasise that while most ECM fungi are vulnerable to climate change, a small subset may benefit—both dynamics that could potentially reshape community composition.

Nevertheless, responses are not uniform. The distribution of *Russula parazurea* (conifer specialist) is projected to expand initially under SSP370 and SSP585 (2041–2070) but decline by 2070–2100, while *Lactarius hepaticus* (conifer specialist) shows the opposite trend, with distribution decline under SSP126 and expansion under higher CO_2_ emissions in the two time periods (Table S6). Similarly, centroid shifts do not always consistently correspond to the magnitude of projected warming, reflecting nonlinear, hump‐shaped responses (Tables S7 and S8). For instance, a species projected to undergo a northeastward shift under SSP126 may not extend further in that direction under SSP370 or SSP585.

### 4.2 Host Specificity and Fungal Responses

Previous studies have shown that the host trees of ECM fungi respond differently to climate change. Broadleaved trees, such as European beech and temperate oak have been projected to shift northeast with a relatively stable or increasing distribution area, while conifers like Scots pine and Norway spruce are expected to lose area, even as their boundaries move northeast under RCP2.6, RCP4.5 and RCP5.8 for 2061‐2080/2090. (Dyderski et al. 2017; Buras and Menzel 2019). These trends are consistent with our host tree projections, where broadleaves fare better than conifers under climate change scenarios (Figure S4; Table S9), and centroids generally move northeast, except for *Q. robur* which is projected to shift southwest (Figures S8–S12).

The distribution area of most studied fungi is projected to reduce under climate change, but the magnitude of this change differs with host specificity. Broadleaf specialists are projected to experience the smallest reduction in range, while conifer specialists are the most vulnerable to climate change (Table 1). This mirrors host tree trends, with broadleaves projected to be ‘winners’ regarding distribution area under climate change, while conifers would be ‘losers’ (Dyderski et al. 2017). ECM fungal generalists, although flexible, appear more strongly tied to conifers than broadleaves in our models (Figure 1). On the other hand, the directional shifts of fungal distributions also vary. Conifer specialists mostly move northeast, about half of broadleaf specialists move southwest, and generalists split between the two (Figure 2; Table S7). These patterns are consistent with the changes of host tree distributions in the future mentioned above. Such links reinforce the critical role of host trees in shaping fungal future distributions and community. However, specificity also implies vulnerability; if broadleaf hosts expand into new regions, they may initially encounter only a subset of their current ECM fungal partners, potentially altering nutrient or C cycling and their growth rates. Generalists may buffer some of these losses, but their dominance could lead to homogenisation of symbiotic networks with potential consequences for forest resilience (Peay et al. 2016; van der Linde et al. 2018).

### 4.3 Co‐Occurrences of ECM Fungi Under Climate Change

**FIGURE 3:**
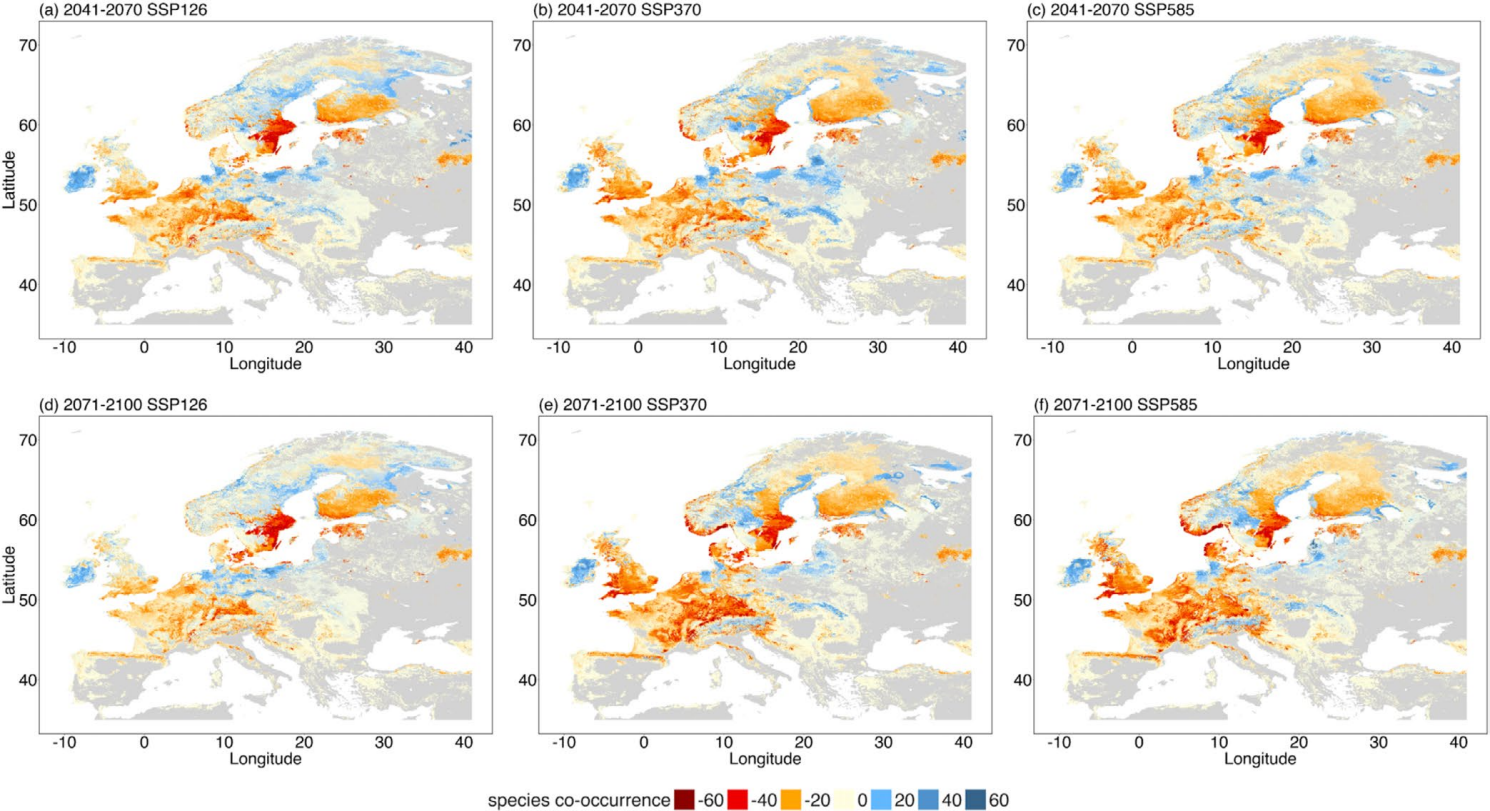
Projected changes in species co‐occurrences per pixel based on ectomycorrhizal fungal binary distribution maps under three SSPs for (a–c) 2041–2070 and (d–f) 2071–2100. Negative values represent the decrease of species co‐occurrence, positive values represent the increase of species co‐occurrence, and zero represents no change in species co‐occurrence.

Co‐occurrence of the 60 modelled fungi is projected to decline sharply across central and northern Europe, including the UK, especially under scenario SSP585 by the end of this century (Figure 3). Areas near Stockholm in Sweden and across Finland are projected to lose nearly all the extant species included in the study in the future, even under low emissions (Figure 3). These shifts suggest major community turnover and diversity losses in key regions. As high ECM fungal diversity has been linked to greater drought resistance and resilience of ecosystems (Liu et al. 2022), projected losses of these species could weaken ecosystem functioning, even if ecosystem stability remains unchanged. However, our analysis is limited to 60 species; others not included may expand into vacated niches, potentially offsetting local diversity losses (Peay et al. 2016). Future research that considers the full ECM fungal community is needed for a more comprehensive assessment. More broadly, declining ECM fungal co‐occurrence suggests that forests may experience reduced symbiont diversity even where tree hosts persist. This could weaken ecosystem resilience to stressors like drought and disturbance, since fewer symbiotic fungi may limit functional complementarity or redundancy. Soil conditions may further restrict recovery; unsuitable pH or nutrient availability could prevent fungi from establishing even in climatically suitable regions, exacerbating mismatches between trees and their symbionts. Unfortunately, we were not able to account for this due to the lack of future modelled soil layers.

### 4.4 Drivers of ECM Fungal Distributions

Both climate variables and host plant related variables have shown an important role in determining ECM fungal distributions (Wollan et al. 2008; Guo et al. 2017; Větrovský et al. 2019; Yu et al. 2021; Qi et al. 2024). In our study, variable importance analyses (Figure 1; Figures S1–S3; Tables S1–S3) showed host trees contributed more to fungal models than climatic predictors, consistent with the obligate symbiosis of ECM fungi, which cannot persist without hosts even in climatically suitable conditions (Smith and Read 2008). Climatic factors such as temperature and precipitation influence fungal richness and composition (Tedersoo et al. 2012; Morgado et al. 2015), but these effects likely act indirectly, via host trees. For example, isothermality strongly influenced conifer specialists, likely because conifer hosts are themselves sensitive to this factor (Huang et al. 2021). Soil pH also emerged as important, with strong effects on conifer‐associated fungi, consistent with host‐driven alteration of soil environments (Rigueiro‐Rodríguez et al. 2011). Together, these findings confirm that host trees are the main factor in shaping ECM fungal distributions, though direct physiological thresholds in fungi remain an open research question.

### 4.5 Limitations and Future Perspectives

This study is the first to project future distributions for comment ECM fungal species across Europe under different climate scenarios and explicitly incorporating host specificity. However, several limitations remain.

First, we relied on GFDL‐ESM4 for future climate projections. This model is recommended as the preferred CMIP6 global climate model (GCM) by the CHELSA database due to its strong performance in reproducing observed climate, making it a robust baseline choice (CHELSA V2.1: Technical specification). But, reliance on a single climate model may underestimate uncertainty because each GCM has distinct biases, climate sensitivities and internal variability. The ISIMIP3b protocol therefore recommends comparison with at least one additional priority model (e.g., MPI‐ESM1‐2‐HR, MRI‐ESM2‐0, IPSL‐CM6A‐LR, UKESM1‐0‐LL) to better represent the uncertainty range in CMIP6 projections (Boyles et al. 2024). Future studies should use bias‐adjusted CHELSA data from multiple GCMs to evaluate the robustness of ECM fungal projections across emission scenarios and key climatic drivers such as temperature and precipitation.

Second, modelled future soil data are unavailable for future projections. Given the strong role of soil pH and N in predicting ECM fungal community (Cox et al. 2010; Tedersoo et al. 2014; van der Linde et al. 2018; Arrauano‐Castilho et al. 2021), the lack of future soil data may influence model projections.

Third, the five dominant tree species in this study may not be the only host trees for some of the studied ECM fungi. For example, *Russula fragilis* and *Russula ochroleuca* were found to colonise roots of *Quercus rubra* and *Picea nigra* , respectively in Poland (Trocha et al. 2012), but these interactions were not recorded in the data set used in our study. Lack of potential host tree distributions, used as predictors in ECM fungal distribution modelling, could influence the modelled distribution maps of ECM fungi. However, information on host trees of ECM fungi is often lacking, limited or unreliable in public databases (e.g., GBIF). The importance of host trees in our results indicates that reliable information about ECM host trees merits consistent recording.

Fourth, species interactions among the studied fungal species were not considered, though they can strongly shape community structure (Koide et al. 2005; Peay et al. 2007) and thus potentially distributions too. Therefore, untangling these interactions, such as by using joint species distribution models, could help improve ECM fungal distribution inferences (Tikhonov et al. 2019).

Finally, dispersal biology remains an unmodelled factor. While many ECM fungi disperse effectively through abundant wind‐borne spores (Peay et al. 2012), others exhibit limited dispersal ranges (Galante et al. 2011) or require animal vectors. Hypogenous and sequestrate fungi, for example, depend on small mammals or other fauna for dispersal (Lilleskov and Bruns 2005; Trappe and Claridge 2005). Such dependence may constrain their ability to track shifting host ranges under climate change, particularly in fragmented landscapes or where animal dispersers decline.

Despite these limitations, our findings highlight the vulnerability of ECM fungi to climate‐driven habitat change and point to important directions for future research. Conifer specialists are projected to lose the largest portions of their current distributions, reflecting sensitivity to both climate and the decline of host tree distributions. Such losses may have ecological consequences for coniferous forest functioning, including nutrient cycling and C storage. Socioeconomically important species are also at risk: *B. edulis*, valued as a food source, is projected to lose ~46% of its range. Endemics such as *Lactarius fluens* (broadleaf specialist), restricted to Europe (only one likely incorrect observation record in Africa in GBIF in the last 50 years), may face heightened conservation risks and warrant closer monitoring. Conversely, some species may benefit; *L. subumbonatus* is predicted to expand its range, suggesting that host generalism or particular functional traits may confer resilience. These contrasting trajectories illustrate that ECM fungi responses are not uniform and that conservation priorities must balance widespread losses with forest management, food security and the stability of symbiotic networks, underscoring the importance of integrating fungi into broader biodiversity, natural capital and ecosystem assessments.

## 5 Conclusion

We projected the future distributions of 60 ECM fungal species under multiple future climate scenarios. Most species are projected to experience substantial distribution contractions, with geographic centroids shifting northeast or southwest for oak associates.

Conifer specialists appear particularly vulnerable compared to broadleaf specialists and host generalists. These findings highlight the central role of host trees in shaping ECM fungal distributions and the urgency of considering fungi in conservation strategies. Understanding the ecological roles of specific ECM fungi in forest ecosystems is essential for predicting the potential impacts of future changes in their distributions. Targeted monitoring of ECM host specialists and evaluation of their conservation status will be essential to understand and manage forests' responses to climate change.

## Author Contributions

**Muyao Qi:** data curation (lead), formal analysis (lead), methodology (lead), writing – original draft (lead). **Martin I. Bidartondo:** data curation (supporting), methodology (supporting), supervision (lead), writing – review and editing (equal). **Laura M. Suz:** data curation (supporting), supervision (lead), writing – review and editing (equal). **C. David L. Orme:** methodology (supporting), supervision (supporting), writing – review and editing (supporting). **Ricardo Arraiano‐Castilho:** writing – review and editing (supporting). **Carolina Tovar:** formal analysis (supporting), methodology (supporting), supervision (lead), writing – review and editing (equal).

## Funding

The authors have nothing to report.

## Conflicts of Interest

The authors declare no conflicts of interest.

## Supporting information


**Data S1:** ece372743‐sup‐0001‐Supinfo.docx.

## Data Availability

Data that support the findings of this study and main codes can be found in the Dryad data repository at https://doi.org/10.5061/dryad.bcc2fqzpp.
