# Supplementary material for: Predicted Effects of Climate Change on Future Distributions of Ectomycorrhizal Fungi
Source: Ecol Evol. 2025 Dec 17;15(12):e72743. doi: 10.1002/ece3.72743 (PMC12711597; doi:10.1002/ece3.72743)
Supplement: Supplementary file 1 — Data S1: ece372743‐sup‐0001‐Supinfo.docx. [file ECE3-15-e72743-s001.docx]

**Supplementary Material**

# Predicted effects of climate change on future distributions of ectomycorrhizal fungi

**i. Host tree distribution modelling**

The distributions of the five dominant ectomycorrhizal (ECM) host trees were modelled based on data from an ECM fungi below-ground survey by van der Linde, et al. (2018). These trees include three broadleaf trees - European beech (*Fagus sylvatica*), pedunculate and sessile oak (*Quercus robur* and *Q. petraea*), and two conifer trees - Norway spruce (*Picea abies*) and Scots pine (*Pinus sylvestris*). The occurrence records for each species were downloaded from GBIF and cleaned following Qi et al. (2024). We selected a set of eight environmental variables, showing low multicollinearity (threshold = 8), and relevant for modelling tree distributions (Saltré et al., 2014; Buras & Menzel, 2019), out of 22 variables that included 19 climate variables downloaded from the CHELSA database (~ 1km spatial resolution) and three variables downloaded from ISRIC (at 250 m spatial resolution with mapped units). These eight environmental variables were: mean diurnal air temperature range (bio2), temperature seasonality (bio4), mean daily mean air temperatures of the wettest quarter (bio8), precipitation seasonality (bio15), mean monthly precipitation amount of the warmest quarter (bio18), mean monthly precipitation amount of the coldest quarter (bio19), soil total nitrogen (sN) and pH (soil depth layer: 5-15cm). These variables were different from the ones used for the ECM fungal distribution models as trees are influenced by different factors (Saltré et al., 2014; Buras & Menzel, 2019).

For consistency with ECM fungal future distribution modelling, the future climate variables used for projecting the future distributions of host trees were derived from the same climate model (GFDL-ESM4 model) for the same time period (2041-2070 and 2071-2100) and under the same three shared socioeconomic pathways (SSP126, SSP370 and SSP585). All the environmental variables, including present and future variables, were resampled at the same resolution and extent as the variables used in ECM fungal modelling.

The current and future distributions of each tree species were ensemble-modelled, including GLM, GBM, RF and Maxent, with the ‘*biomod2*’ R package (Thuiller et al., 2023; parameter setting details in Table S4). In the process, each algorithm was trained with 10,000 randomly generated pseudo-absence points with five-fold cross validation, and the generated model was evaluated by TSS and ROC. For each species, we retained individual models meeting the thresholds of evaluation scores (TSS >= 0.7 and ROC >= 0.8) for building ensemble model. Each produced ensemble model was evaluated by TSS. The future distribution of each species was projected under three different shared socioeconomic pathways (SSP126, SSP370 and SSP585) for both 2041-2070 and 2071-2100 by using the future climate variables and assuming static soil variables. The future binary distribution map of each tree species was converted from its probability distribution map by using maximum TSS threshold (default TSS threshold in *‘biomod2’* R package) and was used as predictor in ECM fungal distribution modelling.

We calculated the relative change in distribution area for each host tree between the future and present (baseline) using the equation: (Area _future_ – Area _baseline_)/ Area _baseline_ × 100%. We also calculated the distribution centroids of these trees under all scenarios (present and future), and mapped the centroids with the ‘*ggplot*2’ R package. Our results showed that the decline of distribution area more likely occurs in conifer trees than broadleaf trees under climate change (Table S8), and most host trees are projected to shift northeast, while only *Q. robur* is projected to move southwest (Fig. S3).

**ii. Comparison of ECM fungal distribution models**

We modelled the current distributions of ECM fungi with two types of ensemble models to test the influence of host tree distributions on ECM fungal distributions: the E-model, using only six abiotic variables; and the EH-model, using the same abiotic variables as the E-model but adding the predicted binary maps of host trees as explanatory variables. The relative importance of each variable and TSS evaluation scores, distribution area and centroids were calculated for each species in both models. Both models used an ensemble model of four algorithms, which were selected from commonly used algorithms (i.e., regression and machine learning) and showed high performance. The four algorithms were GLM, GBM, RF and Maxent. In each run of the two ensemble models, we used randomly generated pseudo-absence data (i.e., 10,000) in modelling for each species based on Barbet-Massin et al.’s (2012) study. In the modelling process for each species, each algorithm was trained with five-fold cross validation, and the generated model was evaluated by TSS and ROC. We retained individual models with TSS values above 0.7 and ROC values above 0.8 for building ensemble model. The produced ensemble model was evaluated by TSS and only those with high evaluation scores (TSS >= 0.7) were used in subsequent analyses (hereafter referred as valid ensemble models; Table S5). We converted continuous maps to binary ones (presence/ absence) by using the maximum TSS threshold. For consistency, the EH-model included the same parameters, evaluation methods and thresholds as the E-model.

We compared the model performance, distribution areas and centroids between E- and EH-model. Our results showed that the EH-model performed significantly better than the E-model for most species (Fig. S1; Table S1-S3). For the EH-model, ensemble models were produced for 63 species, from which 60 species had TSS evaluation scores larger than 0.7 (Fig. S1a). Three species did not have ensemble models due to low evaluation scores (TSS < 0.7 and/or ROC < 0.8) of the individual models (hereafter referred as invalid individual model; Fig. S1a). In the E-model, only 33 species had ensemble models of which 27 had TSS values higher than 0.7 (Fig. S1a). Thirty-three species had invalid individual models (Fig. S1a). The evaluation scores (TSS values) of the EH-model were significantly larger than those of the E-model for the 33 species which had ensemble models in both the E- and EH-model (Fig. S1b). We selected the 27 species, which had valid ensemble models in the E-model, from both E- and EH-models to explore the influence of host tree distributions on ECM fungal distributions. These 27 species included 13 broadleaf specialists, 11 conifer specialists and three host generalists.

In the E-model, climate-related variables had the strongest influence on the model together with pH (Fig. S2a). In the EH-model, the influence of climate variables was much smaller than that of the host trees for most species (Table. S1). The geographical extent of most studied ECM fungal species when including the host (EH-model) is significantly smaller than when using only abiotic variables (E-model) (Figure. S3a).

**Table S1** The relative importance of variables (based on TSS evaluation scores) in E- and EH-model for 27 ECM fungal species that had valid ensemble models in E-model.

| **Species** | **E-model** | | | | | | **EH-model** | | | | | | | | | | **Host spec*** |
| --- | --- | --- | --- | --- | --- | --- | --- | --- | --- | --- | --- | --- | --- | --- | --- | --- | --- |
|  | bio1 | bio3 | bio12 | pH | sN | OCS | bio1 | bio3 | bio12 | pH | sN | OCS | beech | oak | pine | spruce |  |
| *Cortinarius armeniacus* | 0.27 | 0.12 | 0.0086 | 0.52 | 0.07 | 0.01 | 0.22 | 0.084 | 0.017 | 0.26 | 0.017 | 0.028 | NA | NA | 0.071 | 0.3 | C* |
| *Cortinarius biformis* | 0.13 | 0.12 | 0.036 | 0.63 | 0.058 | 0.024 | 0.095 | 0.13 | 0.031 | 0.3 | 0.023 | 0.057 | NA | NA | 0.2 | 0.17 | C |
| *Entoloma caccabus* | 0.29 | 0.023 | 0.29 | 0.22 | 0.089 | 0.082 | 0.0049 | 0.059 | 0.069 | 0.18 | 0.027 | 0.084 | 0.33 | 0.25 | NA | NA | B* |
| *Hygrophorus olivaceoalbus* | 0.24 | 0.2 | 0.0068 | 0.42 | 0.072 | 0.0091 | 0.13 | 0.11 | 0.081 | 0.22 | 0.031 | 0.033 | NA | NA | 0.037 | 0.36 | C |
| *Inocybe*  *assimilata* | 0.45 | 0.18 | 0.22 | 0.077 | 0.071 | 0.0057 | 0.081 | 0.083 | 0.045 | 0.097 | 0.12 | 0.046 | 0.32 | 0.17 | 0.037 | 0.0065 | G* |
| *Lactarius*  *blennius* | 0.63 | 0.086 | 0.21 | 0.031 | 0.024 | 0.022 | 0.13 | 0.014 | 0.016 | 0.021 | 0.011 | 0.021 | 0.79 | NA | NA | NA | B |
| *Lactarius*  *fluens* | 0.6 | 0.033 | 0.28 | 0.014 | 0.042 | 0.029 | 0.14 | 0.01 | 0.042 | 0.016 | 0.011 | 0.026 | 0.55 | 0.2 | NA | NA | B |
| *Lactarius hepaticus* | 0.62 | 0.02 | 0.17 | 0.098 | 0.037 | 0.055 | 0.52 | 0.3 | 0.02 | 0.041 | 0.032 | 0.055 | NA | NA | 0.25 | 0.052 | C |
| *Lactarius*  *pallidus* | 0.52 | 0.073 | 0.28 | 0.023 | 0.077 | 0.023 | 0.069 | 0.019 | 0.042 | 0.0066 | 0.02 | 0.021 | 0.82 | NA | NA | NA | B |
| *Lactarius subdulcis* | 0.7 | 0.013 | 0.19 | 0.024 | 0.046 | 0.029 | 0.15 | 0.02 | 0.011 | 0.015 | 0.02 | 0.026 | 0.47 | 0.29 | NA | NA | B |
| **Species** | **E-model** | | | | | | **EH-model** | | | | | | | | | | **Host spec*** |
|  | bio1 | bio3 | bio12 | pH | sN | OCS | bio1 | bio3 | bio12 | pH | sN | OCS | beech | oak | pine | spruce |  |
| *Lactarius subumbonatus* | 0.29 | 0.47 | 0.016 | 0.11 | 0.082 | 0.028 | 0.23 | 0.41 | 0.016 | 0.13 | 0.084 | 0.068 | NA | 0.056 | NA | NA | B |
| *Russula amethystina* | 0.19 | 0.24 | 0.19 | 0.15 | 0.16 | 0.065 | 0.11 | 0.21 | 0.1 | 0.21 | 0.093 | 0.078 | NA | NA | 0.021 | 0.18 | C |
| *Russula atropurpurea* | 0.72 | 0.019 | 0.12 | 0.046 | 0.094 | 0.0053 | 0.19 | 0.018 | 0.022 | 0.036 | 0.036 | 0.028 | 0.31 | 0.36 | NA | NA | B |
| *Russula*  *aurora* | 0.6 | 0.012 | 0.24 | 0.045 | 0.086 | 0.017 | 0.19 | 0.032 | 0.081 | 0.061 | 0.048 | 0.038 | 0.26 | 0.29 | NA | NA | B |
| *Russula brunneoviolacea* | 0.58 | 0.015 | 0.25 | 0.075 | 0.055 | 0.033 | 0.065 | 0.028 | 0.028 | 0.018 | 0.015 | 0.022 | 0.27 | 0.56 | NA | NA | B |
| *Russula decolorans* | 0.31 | 0.18 | 0.013 | 0.39 | 0.1 | 0.0063 | 0.21 | 0.14 | 0.037 | 0.17 | 0.039 | 0.045 | NA | NA | 0.12 | 0.23 | C |
| *Russula*  *fellea* | 0.63 | 0.058 | 0.21 | 0.044 | 0.03 | 0.02 | 0.14 | 0.026 | 0.013 | 0.032 | 0.008 | 0.032 | 0.52 | 0.23 | NA | NA | B |
| *Russula*  *firmula* | 0.29 | 0.22 | 0.026 | 0.099 | 0.33 | 0.034 | 0.11 | 0.041 | 0.05 | 0.0084 | 0.12 | 0.11 | NA | NA | 0.095 | 0.47 | C |
| *Russula*  *integra* | 0.35 | 0.21 | 0.02 | 0.2 | 0.21 | 0.0038 | 0.25 | 0.1 | 0.037 | 0.06 | 0.043 | 0.025 | NA | NA | 0.048 | 0.44 | C |
| *Russula*  *nobilis* | 0.63 | 0.058 | 0.19 | 0.043 | 0.034 | 0.038 | 0.12 | 0.021 | 0.015 | 0.028 | 0.015 | 0.05 | 0.53 | 0.23 | NA | NA | B |
| **Species** | **E-model** | | | | | | **EH-model** | | | | | | | | | | **Host spec*** |
|  | bio1 | bio3 | bio12 | pH | sN | OCS | bio1 | bio3 | bio12 | pH | sN | OCS | beech | oak | pine | spruce |  |
| *Russula ochroleuca* | 0.66 | 0.026 | 0.23 | 0.018 | 0.014 | 0.05 | 0.16 | 0.012 | 0.031 | 0.021 | 0.02 | 0.071 | 0.36 | 0.2 | 0.013 | 0.11 | G |
| *Russula*  *parazurea* | 0.67 | 0.0097 | 0.24 | 0.045 | 0.021 | 0.019 | 0.11 | 0.029 | 0.03 | 0.041 | 0.012 | 0.036 | 0.27 | 0.47 | NA | NA | B |
| *Russula*  *sardonia* | 0.57 | 0.018 | 0.087 | 0.27 | 0.043 | 0.012 | 0.35 | 0.013 | 0.034 | 0.094 | 0.0093 | 0.015 | NA | NA | 0.48 | NA | C |
| *Russula*  *silvestris* | 0.3 | 0.45 | 0.081 | 0.094 | 0.073 | 0.0014 | 0.035 | 0.099 | 0.018 | 0.086 | 0.037 | 0.017 | 0.67 | 0.022 | 0.0016 | 0.0089 | G |
| *Russula*  *vinosa* | 0.3 | 0.17 | 0.0045 | 0.33 | 0.18 | 0.007 | 0.18 | 0.12 | 0.03 | 0.12 | 0.053 | 0.038 | NA | NA | 0.14 | 0.32 | C |
| *Tylospora fibrillosa* | 0.17 | 0.18 | 0.37 | 0.13 | 0.06 | 0.086 | 0.059 | 0.079 | 0.21 | 0.028 | 0.016 | 0.11 | NA | NA | 0.0088 | 0.49 | C |
| *Xerocomellus pruinatus* | 0.65 | 0.03 | 0.19 | 0.072 | 0.047 | 0.011 | 0.14 | 0.036 | 0.017 | 0.056 | 0.011 | 0.026 | 0.49 | 0.22 | NA | NA | B |

bio1 = mean annual air temperature, bio3 = isothermality, bio12 = annual precipitation amount, pH = soil pH, sN = soil total nitrogen, OCS = soil organic carbon stock, beech = distribution of European beech, oak = distributions of pedunculate and sessile oak, pine = distribution of Scots pine , spruce = distribution of Noway spruce. Host spec* = host specificity, C* = conifer specialists, B* = broadleaf specialists, G* = generalists.

Table S2 Wilcoxon sighed rank test of the influence of host trees distributions on 27 ECM fungal distribution shift (E- and EH-model).

|  | Wilcoxon signed rank test | |
| --- | --- | --- |
| Longitude by model type* | V = 378 | p-value = 2.98e-08 |
| Latitude by model type | V = 1 | p-value = 1.49e-08 |

* model type: E- and EH-model

Table S3 Average contribution of predictors in ECM fungal distribution modelling

| **Predictors** | **Broadleaf specialists** | **Conifer specialists** | **Generalists** |
| --- | --- | --- | --- |
| **bio1** | 12.4% | 17.3% | 14.3% |
| **bio3** | 6.6% | 10.2% | 5.6% |
| **bio12** | 3.1% | 5.6% | 4.5% |
| **pH** | 6.1% | 11.9% | 10.4% |
| **sN*** | 2.9% | 4.2% | 3.0% |
| **OCS*** | 3.9% | 6.4% | 6.0% |
| **beech** | 31.2% | NA | 18.1% |
| **oak** | 33.9% | NA | 11.2% |
| **spruce** | NA | 29.5% | 19.3% |
| **pine** | NA | 14.8% | 7.6% |

* bio1 = mean annual air temperature, bio3 = isothermality, bio12 = annual precipitation amount, pH = soil pH, sN = soil total nitrogen, OCS = soil organic carbon stock, beech = distribution of European beech, oak = distributions of pedunculate and sessile oak, pine = distribution of Scots pine , spruce = distribution of Noway spruce.

**Table S4** Parameters of four algorithms

| **Algorithms*** | **Parameter setting** |
| --- | --- |
| **GLM** | - type = ‘’quadratic, - interaction.level = 0, - myFormula = NULL, - test = ‘AIC’, - family = bionomial (link = ‘logit’), - control = glm.control(epsilon = 1e-8, maxit = 25, trace = FALSE) |
| **GBM** | - distribution = ‘bernoulli’, - n.trees = 2500, - interaction.depth = 7, - n.minobsinnode = 5, - shrinkage = 0.001, - bag.fraction = 0.5, - train.fraction = 1, - cv.folds = 5, - keep.data = FALSE, - verbose = FALSE, - perf.method = ‘cv’, - n.cores = 1 |
| **RF** | - doclassif = TRUE, - ntree = 500, - mtry = ‘default’, - sampsize = NULL, - nodesize = 5, - maxnodes = NULL |
| **Maxent** | - path_to_maxent.jar = getwd()*, - memory_allocated = 512, - background_data_dir = (automatically used pseudo-absences), - maximumbackground = (automatically used maximum number of pseudo-absences), - maximumiterations = 200, - visible = FALSE, - linear = TRUE, - quadratic = TRUE, - produce = TRUE, - threshold = TRUE, - hinge = TRUE, - lq21qptthreshold = 80, - l2lqthreshold = 10, - hingethreshold = 15, - beta_threshold = -1.0, - beta_categorical = -1.0, - beta_lqp = -1.0, - beta_hinge = -1.0, - betamultiplier = 1, - defaultprevalence = 0.5 |

* GLM = generalised linear model, GBM = generalised boosting model, RF = random forest, Maxent = maximum entropy. path_to_maxent.jar = getwd(): a character corresponding to **maxent.jar** file link.

**Table S5** Evaluation scores of ensemble models for studied 66 ECM fungal species

| **Species** | **TSS evaluation scores** | **Host specificity** |
| --- | --- | --- |
| *Amanita excelsa* | 0.812 | generalist |
| *Amanita rubescens* | 0.833 | generalist |
| *Boletus edulis* | 0.845 | generalist |
| ***Boletus pinophilus*** | **0.677** | **conifer** |
| *Byssocorticium atrovirens* | 0.758 | generalist |
| *Cortinarius armeniacus* | 0.783 | conifer |
| *Cortinarius biformis* | 0.756 | conifer |
| *Cortinarius caperatus* | 0.86 | conifer |
| *Cortinarius comptulus* | 0.791 | generalist |
| *Cortinarius diasemospermus* | 0.791 | generalist |
| *Craterellus tubaeformis* | 0.805 | generalist |
| *Elaphomyces granulatus* | 0.741 | generalist |
| *Entoloma caccabus* | 0.791 | broadleaf |
| *Genea hispidula* | 0.759 | broadleaf |
| ***Humaria hemisphaerica*** | **NA** | **generalist** |
| *Hydnotrya tulasnei* | 0.736 | generalist |
| **Species** | **TSS evaluation scores** | **Host specificity** |
| *Hygrophorus olivaceoalbus* | 0.807 | conifer |
| *Hygrophorus pustulatus* | 0.811 | conifer |
| *Imleria badia* | 0.779 | generalist |
| *Inocybe assimilata* | 0.759 | generalist |
| *Inocybe napipes* | 0.792 | generalist |
| *Lactarius blennius* | 0.811 | broadleaf |
| *Lactarius camphoratus* | 0.809 | generalist |
| *Lactarius fluens* | 0.83 | broadleaf |
| *Lactarius hepaticus* | 0.853 | conifer |
| *Lactarius pallidus* | 0.828 | broadleaf |
| *Lactarius quietus* | 0.822 | broadleaf |
| *Lactarius rufus* | 0.828 | conifer |
| *Lactarius subdulcis* | 0.828 | broadleaf |
| *Lactarius subumbonatus* | 0.751 | broadleaf |
| *Lactarius tabidus* | 0.844 | generalist |
| *Otidea leporina* | 0.714 | conifer |
| *Piloderma bicolor* | 0.737 | conifer |
| ***Piloderma olivaceum*** | **0.636** | **conifer** |
| *Pseudocraterellus undulatus* | 0.805 | broadleaf |
| ***Pseudotomentella griseopergamacea*** | **0.665** | **conifer** |
| *Pseudotomentella tristis* | 0.711 | generalist |
| *Russula adusta* | 0.847 | conifer |
| *Russula amethystina* | 0.768 | conifer |
| *Russula atropurpurea* | 0.814 | broadleaf |
| *Russula aurora* | 0.751 | broadleaf |
| *Russula brunneoviolacea* | 0.797 | broadleaf |
| *Russula decolorans* | 0.804 | conifer |
| *Russula fellea* | 0.83 | broadleaf |
| *Russula firmula* | 0.813 | conifer |
| *Russula fragilis* | 0.92 | broadleaf |
| *Russula heterophylla* | 0.737 | broadleaf |
| **Species** | **TSS evaluation scores** | **Host specificity** |
| *Russula integra* | 0.848 | conifer |
| *Russula nobilis* | 0.826 | broadleaf |
| *Russula ochroleuca* | 0.842 | generalist |
| *Russula paludosa* | 0.807 | conifer |
| *Russula parazurea* | 0.831 | broadleaf |
| *Russula romellii* | 0.761 | broadleaf |
| *Russula sardonia* | 0.797 | conifer |
| *Russula silvestris* | 0.772 | generalist |
| *Russula vesca* | 0.825 | generalist |
| *Russula vinosa* | 0.804 | conifer |
| *Scleroderma citrinum* | 0.806 | generalist |
| *Suillus variegatus* | 0.874 | conifer |
| ***Tomentella sublilacina*** | **NA** | **generalist** |
| *Tricholoma portentosum* | 0.822 | conifer |
| ***Tuber puberulum*** | **NA** | **generalist** |
| *Tylopilus felleus* | 0.801 | conifer |
| *Tylospora asterophora* | 0.712 | conifer |
| *Tylospora fibrillosa* | 0.761 | conifer |
| *Xerocomellus pruinatus* | 0.83 | broadleaf |

* NA means there is no ensemble model produced by the four applied algorithms.

**Table S6** Changes of distribution area under future climate scenarios for 60 ECM fungal species in EH-model. The changes were calculated based on the equation: (Area _future_ – Area _baseline_)/ Area _baseline_ × 100%.

| **Species** | **2041-2070**  **SSP126** | **2041-2070**  **SSP370** | **2041-2070**  **SSP585** | **2071-2100**  **SSP126** | **2071-2100**  **SSP370** | **2071-2100**  **SSP585** | **Host specificity** | **Red List**  **Status** |
| --- | --- | --- | --- | --- | --- | --- | --- | --- |
| *Amanita excelsa* | -19.5% | -11.1% | -17.4% | -23.8% | -34.4% | -37.2% | generalist | No describe |
| *Amanita rubescens* | -18.8% | -20.5% | -21.0% | -22.2% | -36.5% | -36.8% | generalist | No describe |
| *Boletus edulis* | -19.3% | -31.2% | -33.0% | -22.2% | -45.8% | -46.4% | generalist | Stable |
| *Byssocorticium atrovirens* | -15.7% | -10.3% | -7.8% | -17.5% | -18.1% | -14.0% | generalist | No describe |
| ***Cortinarius armeniacus*** | **-29.6%** | **-49.8%** | **-51.8%** | **-30.1%** | **-60.5%** | **-64.4%** | conifer | No describe |
| *Cortinarius biformis* | -23.1% | -36.7% | -35.1% | -20.5% | -48.6% | -48.3% | conifer | No describe |
| *Cortinarius caperatus* | -16.6% | -39.2% | -39.2% | -13.7% | -50.5% | -52.8% | conifer | Stable |
| *Cortinarius comptulus* | -31.9% | -42.4% | -40.3% | -33.0% | -53.9% | -53.1% | generalist | No describe |
| *Cortinarius diasemospermus* | -25.2% | -27.2% | -24.3% | -24.8% | -39.2% | -38.6% | generalist | No describe |
| *Craterellus tubaeformis* | -14.4% | -25.9% | -23.8% | -16.4% | -36.9% | -37.3% | generalist | Unknown |
| *Elaphomyces granulatus* | -14.2% | -15.9% | -15.2% | -16.0% | -28.1% | -31.0% | generalist | No describe |
| **Species** | **2041-2070**  **SSP126** | **2041-2070**  **SSP370** | **2041-2070**  **SSP585** | **2071-2100**  **SSP126** | **2071-2100**  **SSP370** | **2071-2100**  **SSP585** | **Host specificity** | **Red List**  **Status** |
| *Entoloma caccabus* | -6.6% | 1.4% | 3.4% | -11.0% | -11.8% | -8.5% | broadleaf | No describe |
| *Genea hispidula* | -0.3% | 9.4% | 9.5% | -2.9% | -0.9% | 5.5% | broadleaf | No describe |
| *Hydnotrya tulasnei* | -16.3% | -22.0% | -25.1% | -19.0% | -42.1% | -42.8% | generalist | No describe |
| *Hygrophorus olivaceoalbus* | -18.3% | -37.1% | -37.4% | -18.6% | -45.4% | -46.5% | conifer | Stable |
| *Hygrophorus pustulatus* | -28.5% | -38.6% | -35.5% | -27.6% | -49.8% | -47.1% | conifer | No describe |
| *Imleria badia* | -20.4% | -16.5% | -19.1% | -24.0% | -40.0% | -37.8% | generalist | Stable |
| *Inocybe assimilata* | -10.5% | -6.7% | -12.4% | -11.6% | -30.9% | -30.9% | generalist | No describe |
| *Inocybe napipes* | -25.8% | -27.3% | -25.1% | -22.8% | -45.2% | -43.1% | generalist | No describe |
| *Lactarius blennius* | -7.4% | -8.0% | -18.4% | -9.2% | -49.4% | -51.7% | broadleaf | No describe |
| *Lactarius camphoratus* | -15.7% | -19.8% | -20.7% | -17.5% | -36.1% | -36.1% | generalist | No describe |
| *Lactarius fluens* | -22.7% | -32.2% | -34.0% | -21.0% | -60.4% | -61.5% | broadleaf | No describe |
| **Species** | **2041-2070**  **SSP126** | **2041-2070**  **SSP370** | **2041-2070**  **SSP585** | **2071-2100**  **SSP126** | **2071-2100**  **SSP370** | **2071-2100**  **SSP585** | **Host specificity** | **Red List**  **Status** |
| ***Lactarius hepaticus*** | **-7.3%** | **5.2%** | **5.9%** | **-7.6%** | **11.2%** | **17.7%** | conifer | No describe |
| *Lactarius pallidus* | -5.8% | -5.5% | -8.6% | -7.6% | -38.5% | -40.1% | broadleaf | No describe |
| *Lactarius quietus* | -16.2% | -17.2% | -20.6% | -15.5% | -37.1% | -33.0% | broadleaf | No describe |
| *Lactarius rufus* | -18.2% | -42.5% | -45.7% | -20.1% | -54.2% | -54.8% | conifer | No describe |
| *Lactarius subdulcis* | -12.2% | -15.6% | -22.2% | -12.8% | -45.8% | -44.5% | broadleaf | No describe |
| ***Lactarius subumbonatus*** | **27.4%** | **31.0%** | **23.5%** | **21.3%** | **22.9%** | **21.0%** | broadleaf | No describe |
| *Lactarius tabidus* | -13.8% | -23.8% | -25.4% | -17.2% | -38.7% | -39.3% | generalist | No describe |
| *Otidea leporina* | -30.2% | -40.2% | -38.9% | -29.3% | -50.2% | -48.4% | conifer | No describe |
| *Piloderma bicolor* | -25.1% | -38.6% | -38.2% | -22.0% | -53.8% | -52.6% | conifer | No describe |
| *Pseudocraterellus undulatus* | -4.9% | 0.8% | 1.8% | -8.8% | -12.1% | -4.9% | broadleaf | No describe |
|  |  |  |  |  |  |  |  |  |
| **Species** | **2041-2070**  **SSP126** | **2041-2070**  **SSP370** | **2041-2070**  **SSP585** | **2071-2100**  **SSP126** | **2071-2100**  **SSP370** | **2071-2100**  **SSP585** | **Host specificity** | **Red List**  **Status** |
| *Pseudotomentella tristis* | -19.6% | -29.2% | -29.6% | -21.1% | -45.9% | -45.9% | generalist | No describe |
| *Russula adusta* | -19.1% | -27.7% | -27.2% | -17.3% | -43.2% | -41.7% | conifer | No describe |
| *Russula amethystina* | -12.3% | -17.6% | -20.0% | -10.8% | -34.3% | -37.0% | conifer | No describe |
| *Russula atropurpurea* | -4.5% | 1.0% | -9.1% | -8.9% | -28.7% | -29.2% | broadleaf | No describe |
| *Russula aurora* | -8.4% | -2.9% | -7.8% | -10.1% | -26.9% | -25.2% | broadleaf | No describe |
| *Russula brunneoviolacea* | -16.7% | -17.5% | -16.5% | -15.4% | -42.3% | -39.2% | broadleaf | No describe |
| *Russula decolorans* | -27.6% | -47.9% | -49.8% | -25.0% | -57.8% | -60.3% | conifer | Stable |
| *Russula fellea* | -13.6% | -17.5% | -25.7% | -15.4% | -50.1% | -52.6% | broadleaf | No describe |
| *Russula firmula* | -25.1% | -40.0% | -41.2% | -25.3% | -50.4% | -51.9% | conifer | No describe |
| *Russula fragilis* | -1.4% | 0.4% | -3.8% | -7.1% | -17.1% | -14.8% | broadleaf | No describe |
| *Russula heterophylla* | -11.2% | -20.9% | -24.1% | -11.7% | -44.3% | -46.2% | broadleaf | No describe |
| **Species** | **2041-2070**  **SSP126** | **2041-2070**  **SSP370** | **2041-2070**  **SSP585** | **2071-2100**  **SSP126** | **2071-2100**  **SSP370** | **2071-2100**  **SSP585** | **Host specificity** | **Red List**  **Status** |
| *Russula integra* | -23.2% | -31.1% | -29.2% | -22.3% | -38.4% | -38.1% | conifer | No describe |
| *Russula nobilis* | -7.3% | -3.3% | -11.6% | -11.0% | -32.5% | -33.4% | broadleaf | No describe |
| *Russula ochroleuca* | -20.9% | -24.7% | -21.6% | -17.7% | -48.8% | -43.0% | generalist | No describe |
| *Russula paludosa* | -22.2% | -45.5% | -48.6% | -24.4% | -54.8% | -55.6% | conifer | Stable |
| *Russula parazurea* | -1.2% | 3.5% | 3.0% | -5.4% | -18.0% | -9.4% | broadleaf | No describe |
| *Russula romellii* | -15.7% | -18.9% | -24.4% | -19.1% | -45.9% | -47.5% | broadleaf | No describe |
| *Russula sardonia* | -12.6% | -7.2% | -5.5% | -10.1% | -4.8% | -2.5% | conifer | No describe |
| *Russula silvestris* | 0.8% | 9.9% | -0.3% | -3.4% | -16.9% | -20.2% | generalist | No describe |
| *Russula vesca* | -14.5% | -17.4% | -21.5% | -16.2% | -34.6% | -35.6% | generalist | Stable |
| ***Russula vinosa*** | **-28.0%** | **-50.4%** | **-53.1%** | **-28.7%** | **-59.2%** | **-62.0%** | conifer | Stable |
| *Scleroderma citrinum* | -8.4% | 1.1% | -3.2% | -11.9% | -16.6% | -13.6% | generalist | No describe |
| ***Suillus variegatus*** | **0.2%** | **-13.0%** | **-11.3%** | **0.003%** | **-25.3%** | **-21.3%** | conifer | Stable |
| **Species** | **2041-2070**  **SSP126** | **2041-2070**  **SSP370** | **2041-2070**  **SSP585** | **2071-2100**  **SSP126** | **2071-2100**  **SSP370** | **2071-2100**  **SSP585** | **Host specificity** | **Red List**  **Status** |
| *Tricholoma portentosum* | -19.0% | -31.9% | -27.8% | -20.2% | -38.0% | -35.3% | conifer | No describe |
| *Tylopilus felleus* | -22.3% | -33.6% | -31.6% | -21.7% | -47.0% | -46.3% | conifer | No describe |
| *Tylospora asterophora* | -22.6% | -34.3% | -34.9% | -21.9% | -47.2% | -48.2% | conifer | No describe |
| *Tylospora fibrillosa* | -10.3% | -25.9% | -28.1% | -11.3% | -37.9% | -37.1% | conifer | No describe |
| ***Xerocomellus pruinatus*** | **-18.4%** | **-24.5%** | **-30.2%** | **-19.5%** | **-60.8%** | **-60.5%** | broadleaf | No describe |

**Table S7**  The number of the 60 studied species distribution shift in four directions under six future climate scenarios.

| Scenarios | | Northeast | | | Northwest | | | Southwest | | | Southeast | | |
| --- | --- | --- | --- | --- | --- | --- | --- | --- | --- | --- | --- | --- | --- |
|  |  | B^*^ | C^*^ | G^*^ | B | C | G | B | C | G | B | C | G |
| 2041  \|  2070 | SSP126 | 8 | 17 | 8 | 2 | 1 | 1 | 9 | 4 | 10 | 0 | 0 | 0 |
|  | SSP370 | 10 | 15 | 7 | 0 | 2 | 2 | 9 | 4 | 9 | 0 | 1 | 1 |
|  | SSP585 | 8 | 15 | 7 | 2 | 1 | 2 | 9 | 5 | 9 | 1 | 1 | 0 |
| 2071  \|  2100 | SSP126 | 6 | 16 | 5 | 2 | 1 | 4 | 10 | 4 | 10 | 1 | 1 | 0 |
|  | SSP370 | 8 | 16 | 10 | 3 | 1 | 0 | 8 | 4 | 9 | 0 | 1 | 0 |
|  | SSP585 | 8 | 16 | 9 | 3 | 0 | 1 | 8 | 5 | 9 | 0 | 1 | 0 |

*B: broadleaf specialists (19 in total); C: conifer specialists (22 in total); G: generalists (19 in total)

**Table S8** Angles in centroids between current distribution and projected distributions under climate change scenarios for 60 species.

| **Species** | **2041-2070**  **SSP126** | **2041-2070**  **SSP370** | **2041-2070**  **SSP585** | **2071-2100**  **SSP126** | **2071-2100**  **SSP370** | **2071-2100**  **SSP585** | **Host specificity** |
| --- | --- | --- | --- | --- | --- | --- | --- |
| *Amanita excelsa* | 33.3 | 21.3 | 46.0 | 97.2 | 47.9 | 61.0 | generalist |
| *Amanita rubescens* | 80.2 | 53.1 | 80.9 | 101.9 | 62.9 | 63.9 | generalist |
| *Boletus edulis* | 65.5 | 54.8 | 84.2 | 82.6 | 63.1 | 69.4 | generalist |
| *Byssocorticium atrovirens* | 233.6 | 238.8 | 235.4 | 229.0 | 236.4 | 237.7 | generalist |
| *Cortinarius armeniacus* | 58.4 | 60.4 | 63.2 | 60.4 | 61.0 | 62.1 | conifer |
| *Cortinarius biformis* | 83.1 | 112.9 | 159.3 | 85.7 | 169.5 | 195.8 | conifer |
| *Cortinarius caperatus* | 92.7 | 155.5 | 196.5 | 94.9 | 212.9 | 225.4 | conifer |
| *Cortinarius comptulus* | 228.5 | 237.5 | 237.9 | 229.1 | 235.7 | 238.4 | generalist |
| *Cortinarius diasemospermus* | 214.9 | 224.8 | 224.4 | 213.0 | 216.1 | 217.8 | generalist |
| *Craterellus tubaeformis* | 86.9 | 105.4 | 144.7 | 98.4 | 76.4 | 97.1 | generalist |
| *Elaphomyces granulatus* | 214.4 | 227.2 | 223.3 | 210.4 | 184.4 | 204.8 | generalist |
| *Entoloma caccabus* | 216.8 | 230.0 | 225.3 | 204.2 | 169.6 | 105.3 | broadleaf |
| *Genea hispidula* | 4.2 | 9.4 | 3.9 | 359.5 | 20.4 | 17.4 | broadleaf |
| *Hydnotrya tulasnei* | 72.4 | 66.0 | 77.2 | 79.3 | 71.7 | 73.0 | generalist |
| *Hygrophorus olivaceoalbus* | 59.1 | 60.7 | 63.4 | 61.6 | 60.4 | 61.5 | conifer |
| *Hygrophorus pustulatus* | 67.0 | 66.6 | 71.2 | 71.1 | 66.7 | 70.4 | conifer |
| *Imleria badia* | 236.9 | 245.6 | 238.4 | 232.4 | 233.6 | 235.5 | generalist |
| *Inocybe assimilate* | 223.6 | 230.4 | 223.7 | 221.3 | 213.9 | 210.2 | generalist |
| *Inocybe napipes* | 128.9 | 105.4 | 116.0 | 142.4 | 82.1 | 82.2 | generalist |
| **Species** | **2041-2070**  **SSP126** | **2041-2070**  **SSP370** | **2041-2070**  **SSP585** | **2071-2100**  **SSP126** | **2071-2100**  **SSP370** | **2071-2100**  **SSP585** | **Host specificity** |
| *Lactarius blennius* | 31.6 | 23.9 | 37.1 | 49.2 | 42.3 | 47.8 | broadleaf |
| *Lactarius camphoratus* | 60.9 | 51.0 | 57.7 | 67.5 | 55.3 | 55.0 | generalist |
| *Lactarius fluens* | 140.7 | 67.5 | 92.6 | 165.7 | 100.0 | 92.4 | broadleaf |
| *Lactarius hepaticus* | 223.3 | 244.0 | 241.3 | 225.2 | 338.3 | 355.5 | conifer |
| *Lactarius pallidus* | 21.5 | 23.4 | 30.1 | 33.7 | 33.5 | 39.6 | broadleaf |
| *Lactarius quietus* | 48.6 | 48.2 | 52.7 | 53.8 | 60.8 | 55.5 | broadleaf |
| *Lactarius rufus* | 64.0 | 64.7 | 68.8 | 65.5 | 65.5 | 67.2 | conifer |
| *Lactarius subdulcis* | 117.9 | 58.5 | 91.5 | 139.8 | 104.2 | 93.7 | broadleaf |
| *Lactarius subumbonatus* | 264.6 | 265.8 | 263.4 | 264.0 | 264.5 | 264.7 | broadleaf |
| *Lactarius tabidus* | 57.1 | 51.6 | 55.5 | 59.7 | 53.4 | 51.7 | generalist |
| *Otidea leporine* | 70.5 | 72.5 | 74.0 | 71.5 | 72.4 | 72.7 | conifer |
| *Piloderma bicolor* | 49.3 | 45.6 | 44.6 | 50.0 | 49.3 | 41.5 | conifer |
| *Pseudocraterellus undulatus* | 224.1 | 223.6 | 223.9 | 222.9 | 219.1 | 220.1 | broadleaf |
| *Pseudotomentella tristis* | 232.6 | 234.4 | 233.3 | 230.2 | 229.8 | 231.1 | generalist |
| *Russula adusta* | 228.0 | 231.4 | 230.7 | 228.1 | 227.6 | 228.6 | conifer |
| *Russula amethystina* | 0.3 | 352.0 | 346.7 | 351.9 | 19.1 | 20.3 | conifer |
| *Russula atropurpurea* | 213.6 | 221.8 | 212.7 | 211.9 | 206.1 | 203.1 | broadleaf |
| *Russula aurora* | 234.0 | 243.6 | 235.8 | 229.1 | 229.6 | 227.7 | broadleaf |
| *Russula brunneoviolacea* | 226.7 | 234.2 | 232.4 | 224.4 | 203.7 | 209.6 | broadleaf |
| *Russula decolorans* | 60.9 | 62.4 | 64.6 | 62.1 | 62.9 | 63.6 | conifer |
| **Species** | **2041-2070**  **SSP126** | **2041-2070**  **SSP370** | **2041-2070**  **SSP585** | **2071-2100**  **SSP126** | **2071-2100**  **SSP370** | **2071-2100**  **SSP585** | **Host specificity** |
| *Russula fellea* | 20.5 | 19.0 | 34.9 | 33.9 | 41.2 | 49.5 | broadleaf |
| *Russula formula* | 53.9 | 51.5 | 53.0 | 55.4 | 51.9 | 51.2 | conifer |
| *Russula fragilis* | 43.1 | 48.4 | 51.9 | 48.5 | 60.4 | 55.5 | broadleaf |
| *Russula heterophylla* | 243.7 | 242.8 | 241.6 | 242.1 | 237.3 | 235.3 | broadleaf |
| *Russula integra* | 49.4 | 47.2 | 46.0 | 50.1 | 49.7 | 47.2 | conifer |
| *Russula nobilis* | 225.9 | 231.9 | 224.3 | 222.4 | 220.5 | 218.3 | broadleaf |
| *Russula ochroleuca* | 194.3 | 199.5 | 200.2 | 195.0 | 187.0 | 188.5 | generalist |
| *Russula paludosa* | 58.9 | 60.0 | 62.7 | 60.8 | 59.8 | 60.5 | conifer |
| *Russula parazurea* | 202.0 | 203.6 | 204.4 | 202.2 | 197.7 | 197.9 | broadleaf |
| *Russula romellii* | 42.1 | 35.8 | 45.7 | 66.8 | 45.2 | 51.8 | broadleaf |
| *Russula sardonia* | 51.5 | 49.3 | 45.1 | 52.2 | 45.8 | 44.9 | conifer |
| *Russula silvestris* | 242.0 | 247.6 | 242.7 | 239.2 | 241.7 | 241.4 | generalist |
| *Russula vesca* | 41.8 | 30.6 | 37.4 | 47.2 | 42.2 | 41.1 | generalist |
| *Russula vinosa* | 57.4 | 58.1 | 61.5 | 59.6 | 58.1 | 58.9 | conifer |
| *Scleroderma citrinum* | 256.5 | 347.1 | 308.6 | 220.7 | 17.7 | 24.7 | generalist |
| *Suillus variegatus* | 55.7 | 54.6 | 54.2 | 55.9 | 54.6 | 53.1 | conifer |
| *Tricholoma portentosum* | 52.8 | 52.3 | 46.2 | 55.4 | 49.9 | 46.6 | conifer |
| *Tylopilus felleus* | 236.1 | 238.8 | 238.2 | 234.9 | 234.9 | 237.3 | conifer |
| *Tylospora asterophora* | 49.9 | 49.1 | 48.5 | 49.9 | 50.7 | 50.2 | conifer |
| **Species** | **2041-2070**  **SSP126** | **2041-2070**  **SSP370** | **2041-2070**  **SSP585** | **2071-2100**  **SSP126** | **2071-2100**  **SSP370** | **2071-2100**  **SSP585** | **Host specificity** |
| *Tylospora fibrillosa* | 255.4 | 251.6 | 245.5 | 239.8 | 258.1 | 250.6 | conifer |
| *Xerocomellus pruinatus* | 4.6 | 15.3 | 37.4 | 187.5 | 42.8 | 53.9 | broadleaf |

Angles between 0 to 90 represent a shift of distribution centroid to northeast, between 90 to 180 represent a shift to northwest, between 180 to 270 represent a shift to southwest and between 270 to 360 represent a shift to southeast.

**Table S9** The current distribution area (km^2^) and the distribution area change ratio under climate change of the five ectomycorrhizal fungal host trees.

| Host trees | | **European beech** | **Sessile and pedunculate oak** | | **Scots pine** | **Norway spruce** |
| --- | --- | --- | --- | --- | --- | --- |
|  |  | *F. sylvatica* | *Q. petraea* | *Q. robur* | *P. sylvestris* | *P. abies* |
| Current distribution area | | 1772246 | 1137561 | 1341378 | 1523407 | 1386108 |
| The change ratio of distribution area under climate change | **2041-2070** |  |  |  |  |  |
|  | SSP126 | 3.7% | 2.0% | -28.8% | -39.3% | -36.3% |
|  | SSP370 | 16.5% | 6.8% | -21.2% | -50.6% | -49.2% |
|  | SSP585 | 8.9% | 5.6% | -19.8% | -49.9% | -46.4% |
|  | **2071-2100** |  |  |  |  |  |
|  | SSP126 | -3.4% | -0.5% | -25.4% | -38.6% | -36.5% |
|  | SSP370 | -3.8% | -16.7% | -45.2% | -65.4% | -60.2% |
|  | SSP585 | -4.6% | -14.1% | -40.5% | -65.9% | -58.2% |


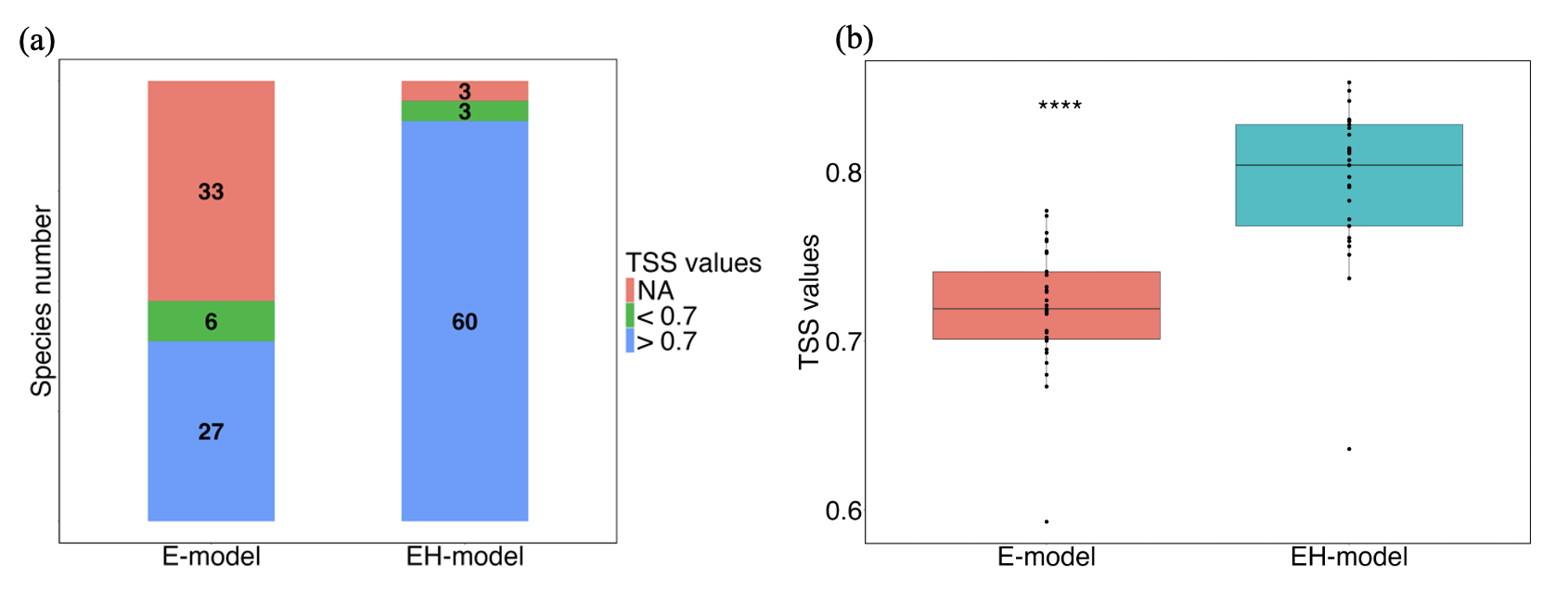


****: p <= 0.0001

**Figure S1** Comparison of the TSS model evaluation scores between the E- and EH-models for all 66 ectomycorrhizal (ECM) fungal species. (a) Number of valid ensemble models from E- and EH-model. The blue represents the number of ECM fungal species which had valid ensemble model (TSS value >= 0.7), the green represents the number of ECM fungal species which had invalid ensemble model (TSS value < 0.7), and the red represents the number of species which did not have an ensemble model (TSS value = NA); (b) TSS values of 33 species which produced ensemble models in both E- and EH- models.


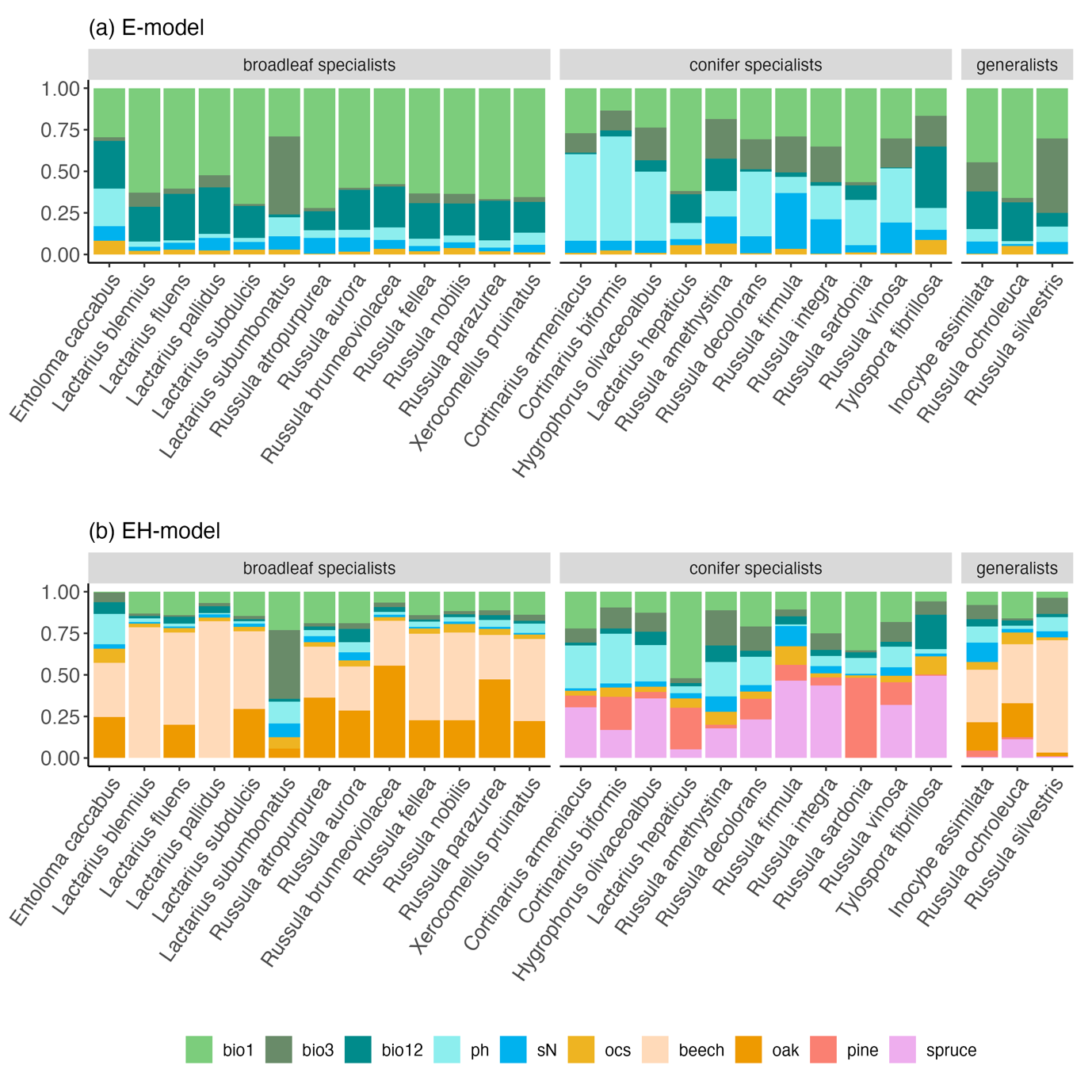


**Figure S2**  Relative importance of each variable (0 to 1) included in the a) E- and b) EH-models based on the host specificity of 27 ECM fungi that had valid ensemble models in the E-model.

bio1 = mean annual air temperature, bio3 = isothermality, bio12 = annual precipitation amount, ph = soil pH, sN = soil total nitrogen, ocs = soil organic carbon stock, beech = distribution of European beech, oak = distributions of pedunculate and sessile oak, pine = distribution of Scots pine, spruce = distribution of Norway spruce.


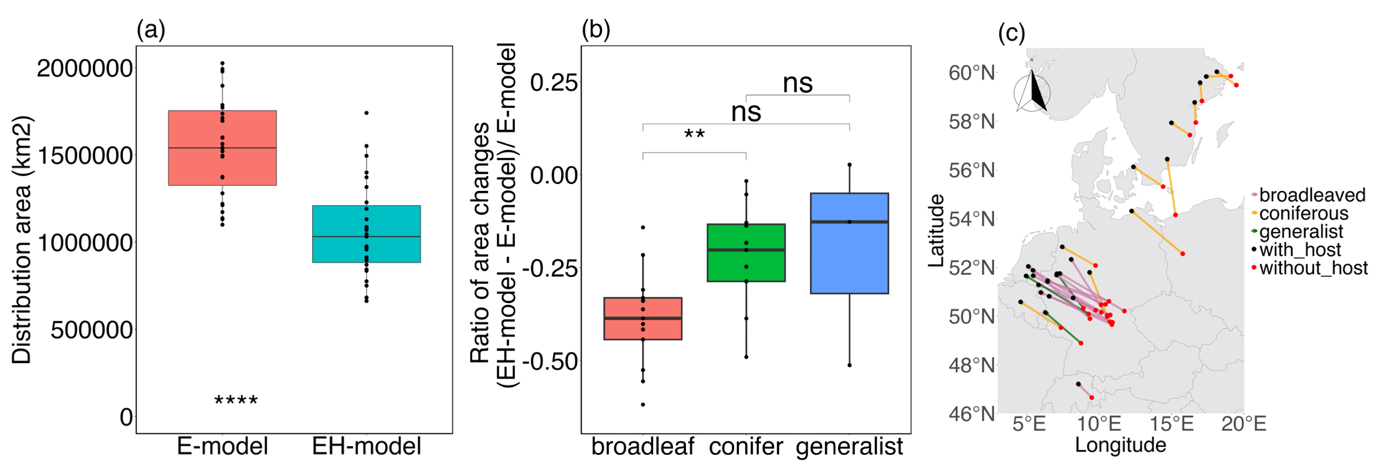


**Figure S3** Comparison between E-model and EH-model. (a) Distribution area of the 27 species which had valid ensemble models for the E- and EH-model. Differences of distribution area were tested with Wilcoxon sign-rank test. (b) Ratio of distribution area changes for broadleaf specialists, conifer specialists and generalists. Differences of the ratio among the three groups were tested with Wilcoxon sign sum test. (c) The distribution centroids of the 27 species on map. The arrows connect the pairs of distribution centroids (from E- and EH-model) for each species.


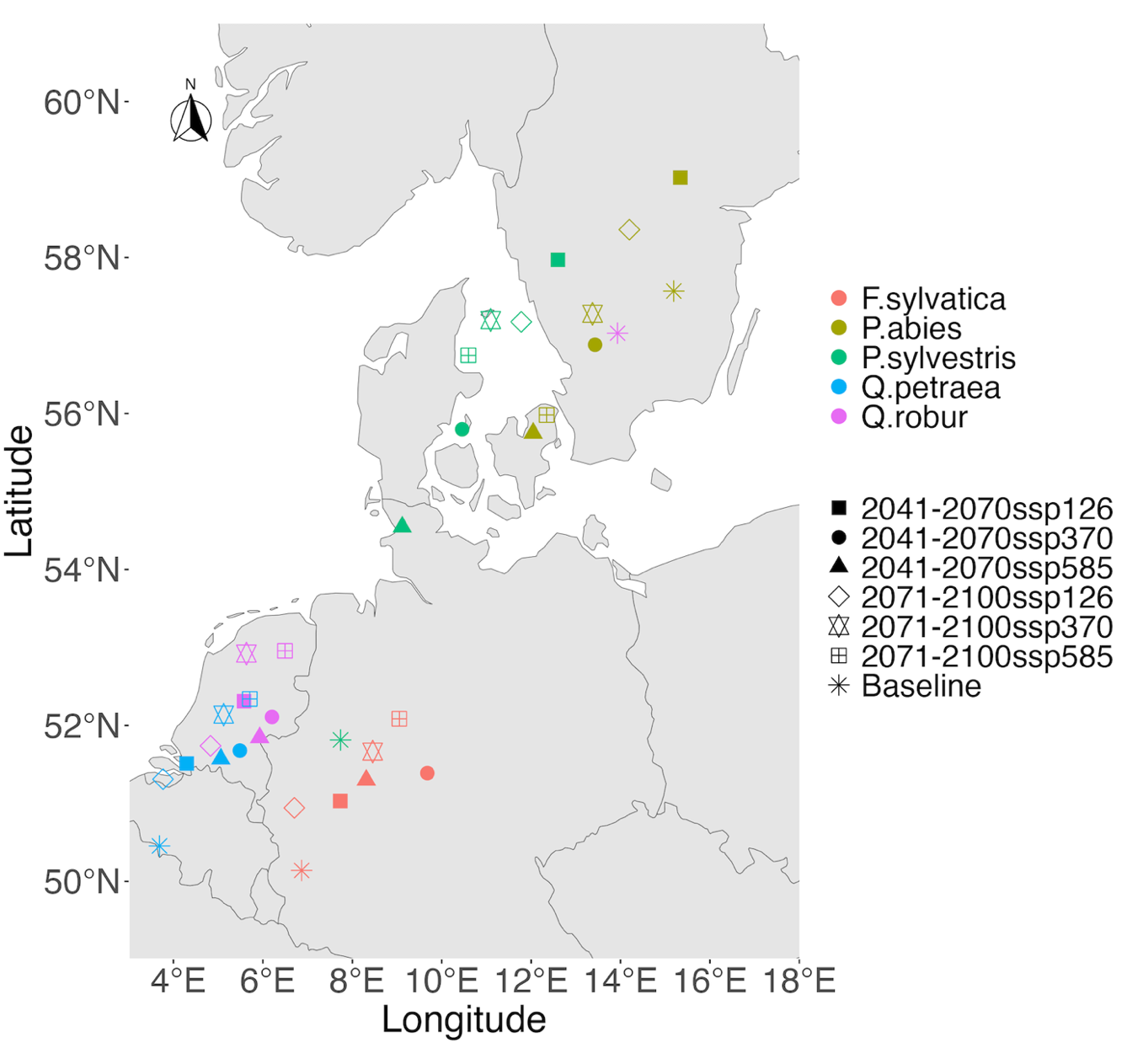


**Figure S4** The centroids of current distributions (baseline) and future distributions for the selected five host trees.


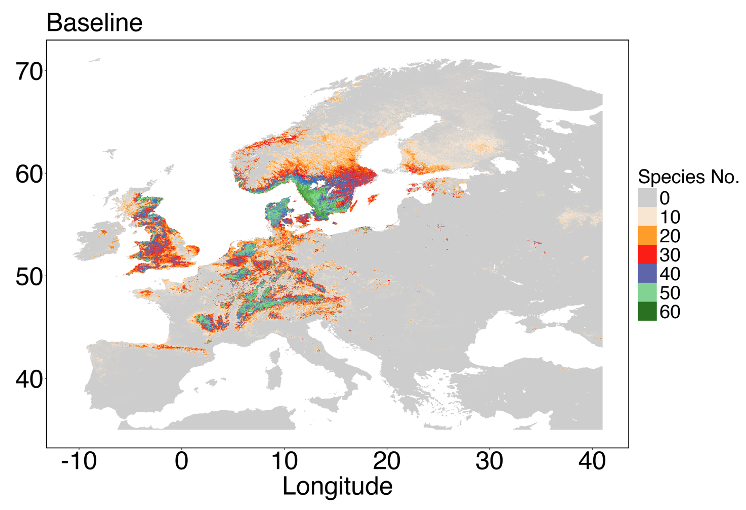


**Figure S5** The current co-occurrence of studied 60 ectomycorrhizal fungal species in each pixel in studied area.


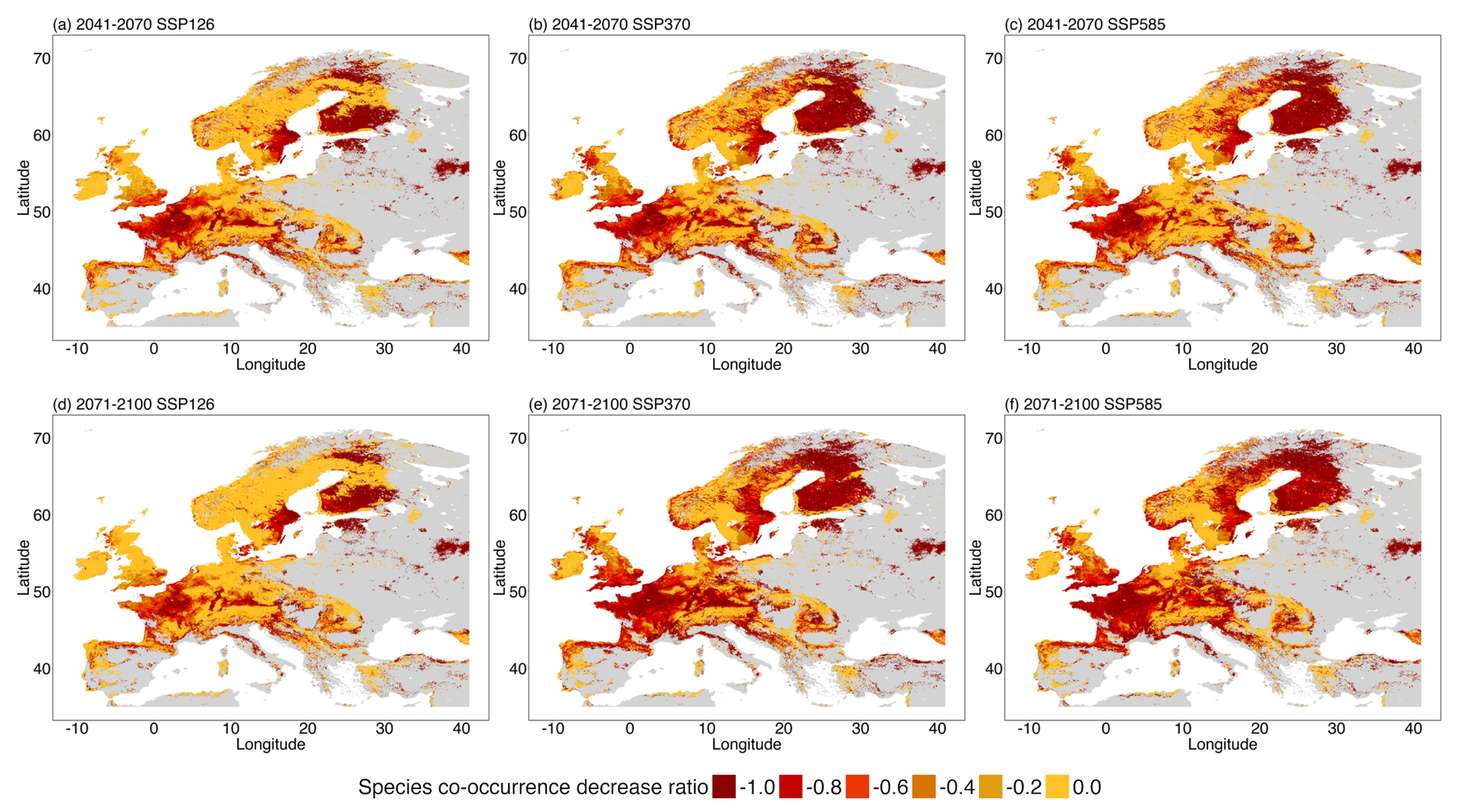


**Figure S6** Predicted the decrease of species co-occurrences in each pixel under three socioeconomic pathways for (a-c) 2041-2070 and (d-f) 2071-2100 for the 60 ECM fungal species.


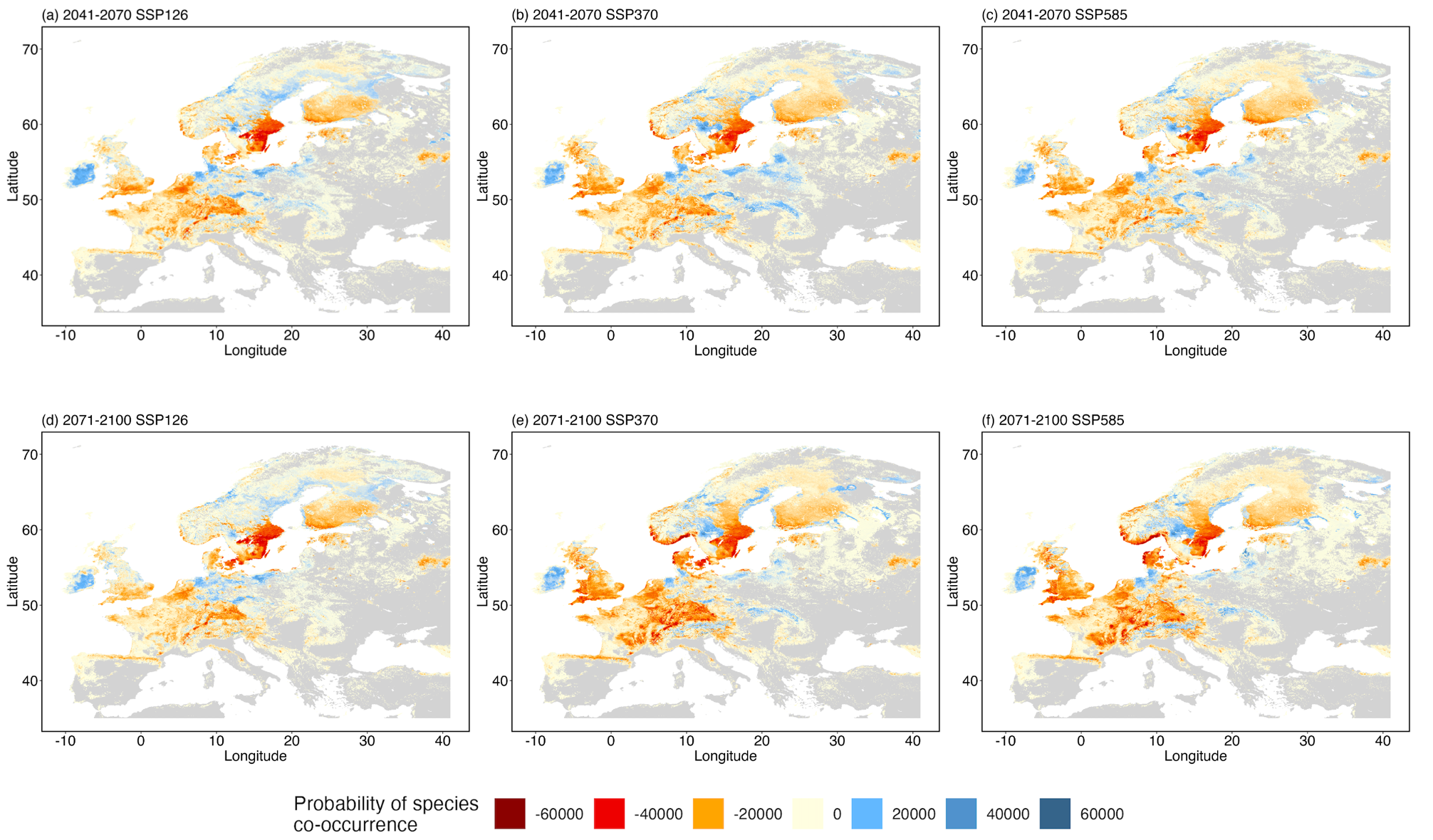


**Figure S7** Predicted the changes of species co-occurrences in each pixel under three socioeconomic pathways for (a-c) 2041-2070 and (d-f) 2071-2100 for the 60 ECM fungal species by using their probability distributions which used the probability distributions of host trees as predictor.


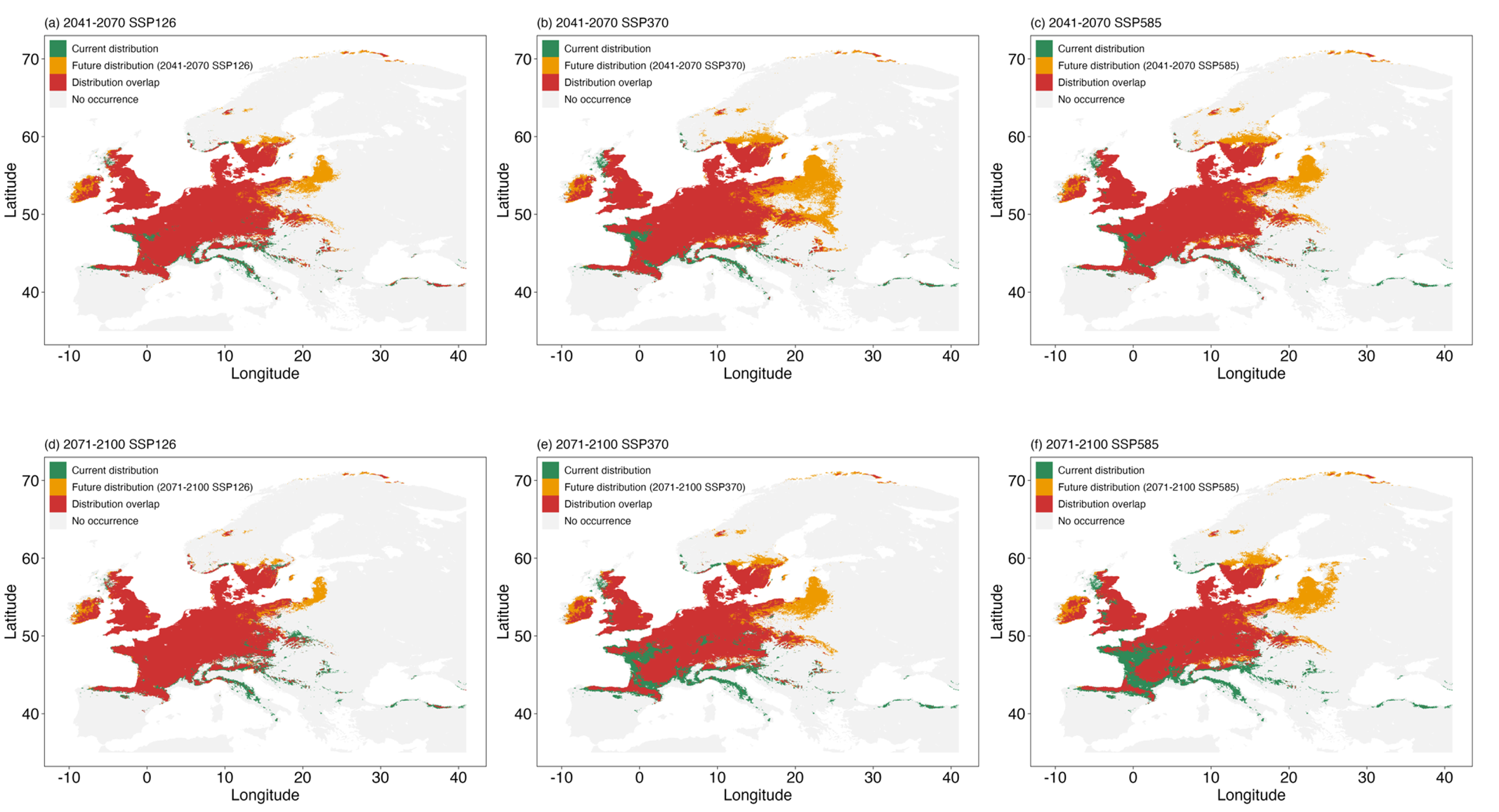


**Figure S8** The distribution changes of European beech under three climate scenarios in 2041-2070. The green parts represent the current distributions and the orange part represent the future distribution. The red parts represent the distribution in both present and future.


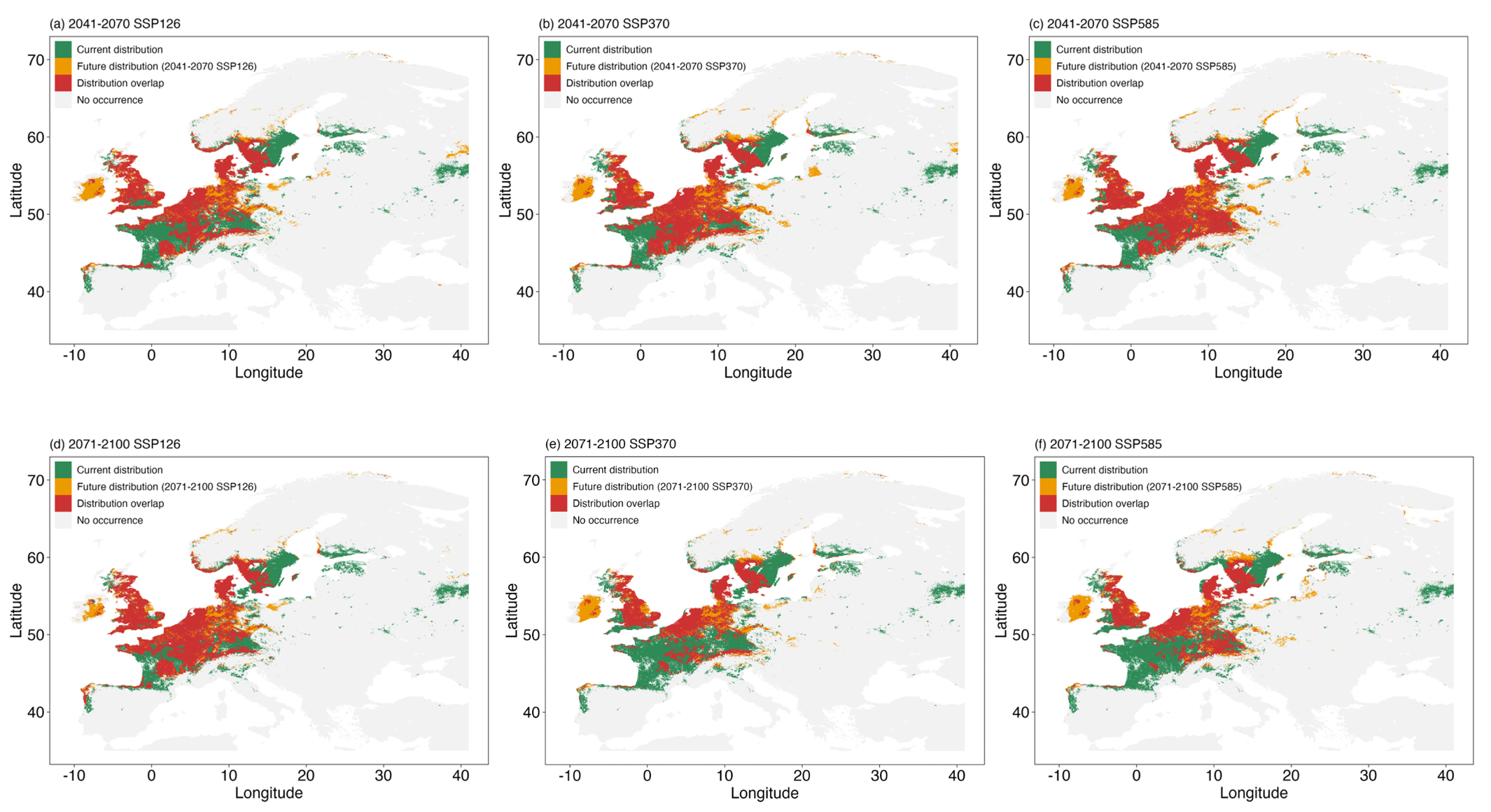


**Figure S9** The distribution changes of Pedunculate oak under three climate scenarios in 2041-2070. The green parts represent the current distributions and the orange part represent the future distribution. The red parts represent the distribution in both present and future.


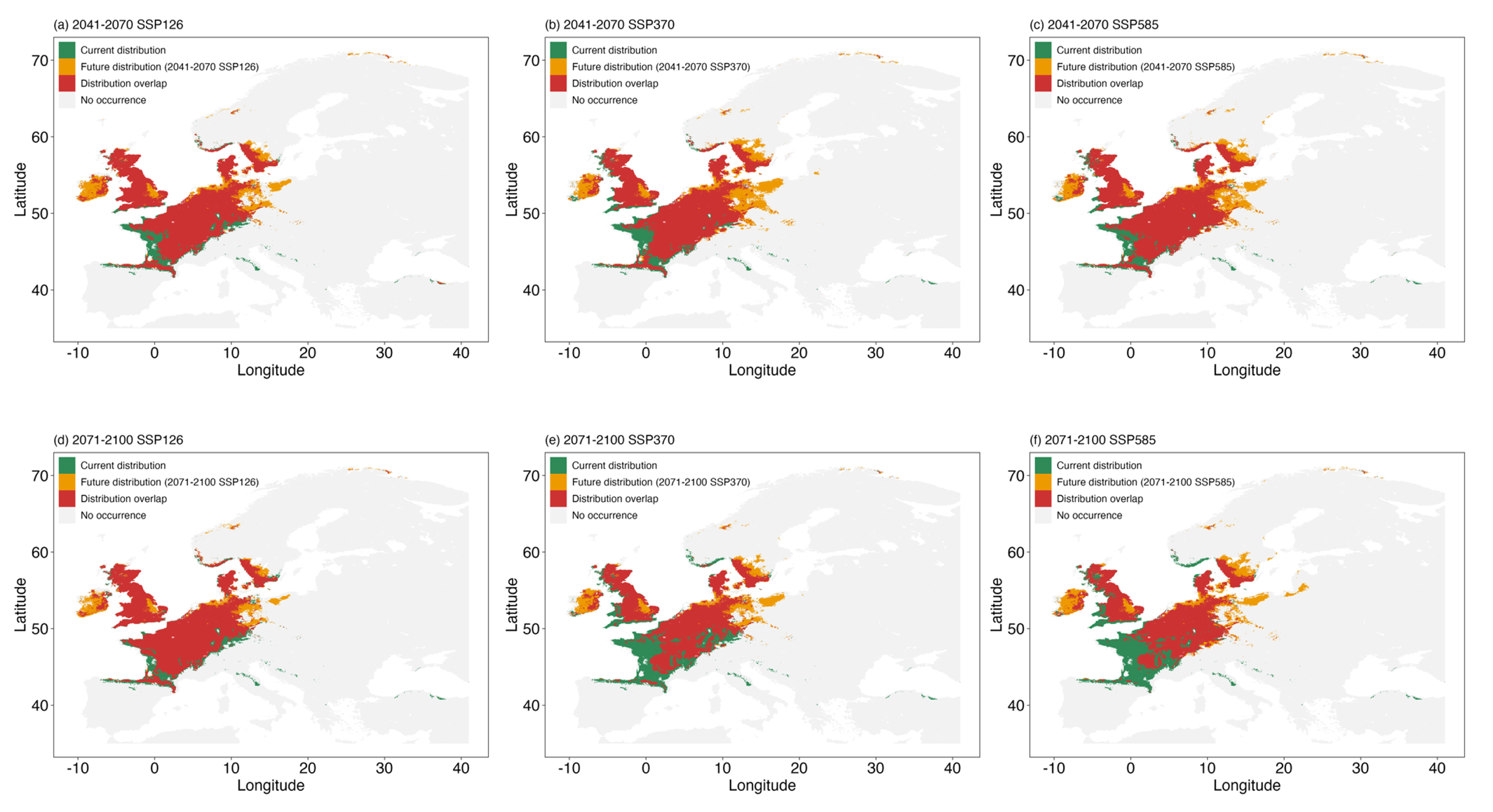


**Figure S10** The distribution changes of Sessile oak under three climate scenarios in 2041-2070. The green parts represent the current distributions and the orange part represent the future distribution. The red parts represent the distribution in both present and future.


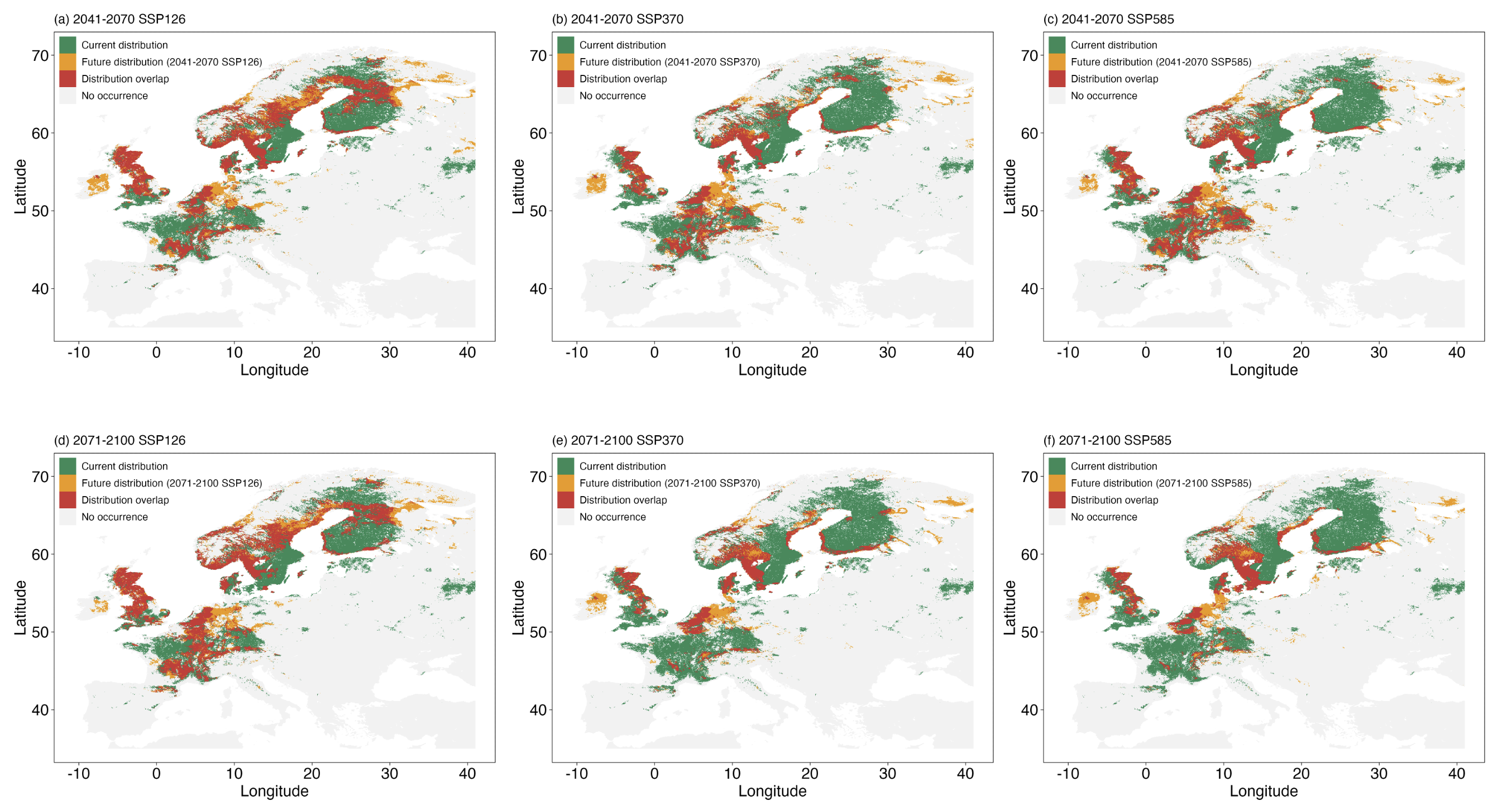


**Figure S11** The distribution changes of Scots pine under three climate scenarios in 2041-2070. The green parts represent the current distributions and the orange part represent the future distribution. The red parts represent the distribution in both present and future.


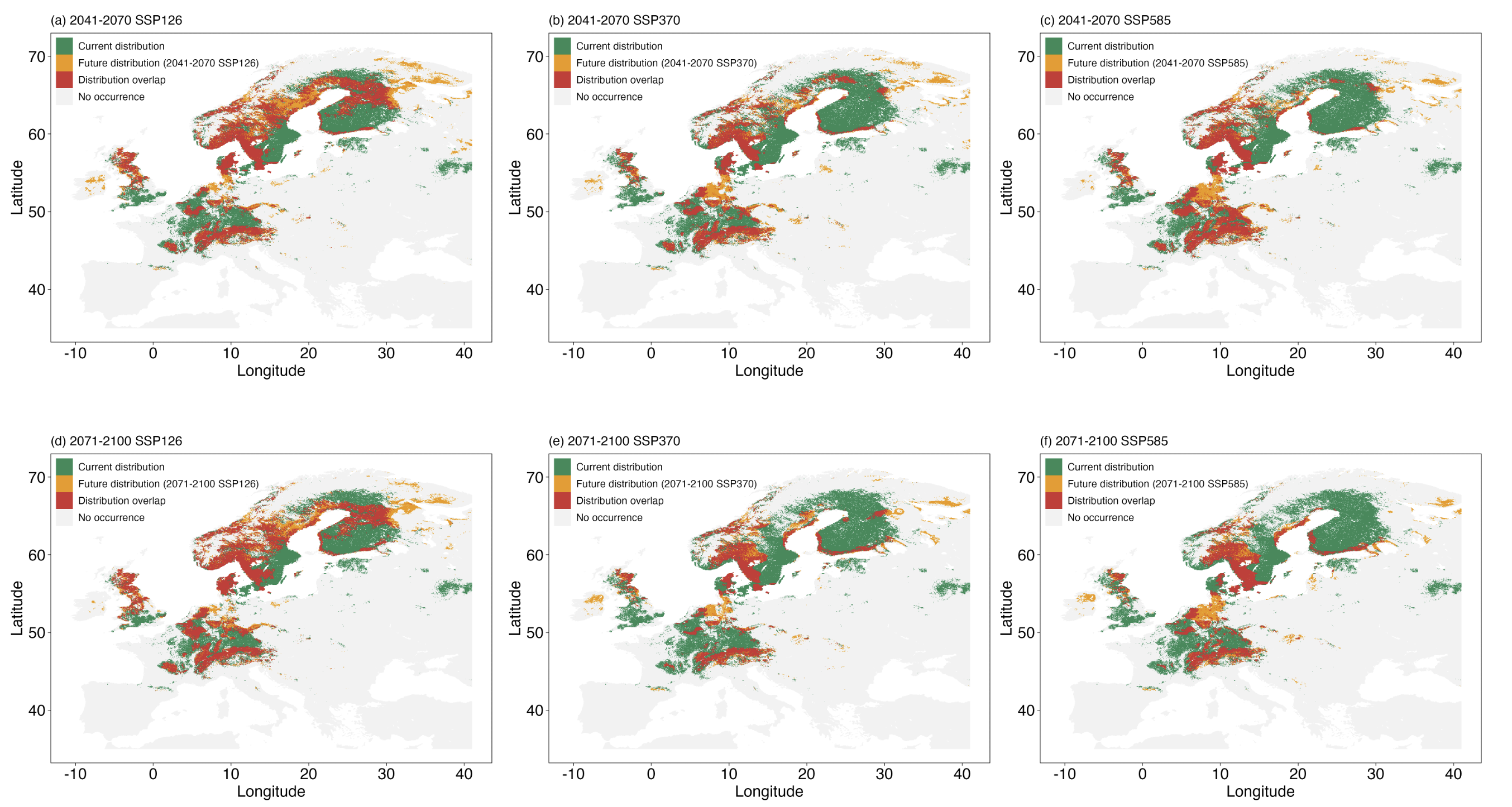


**Figure S12** The distribution changes of Norway spruce under three climate scenarios in 2041-2070. The green parts represent the current distributions and the orange part represent the future distribution. The red parts represent the distribution in both present and future.

**References**

Buras, A. & Menzel, A. (2019) Projecting tree species composition changes of European forests for 2061-2090 under RCP 4.5 and RCP 8.5 Scenarios. *Frontiers in Plant Science*. 9, 1986. doi: 10.3389/fpls.2018.01986.

Qi, M., Suz, L.M., Bidartondo, M.I., Orme, D.L., Delhaye, G., Opeanshaw, I. & Tovar, C. (2024) Fruitbody and root data infer different environmental niches for ectomycorrhizal fungi. *Journal of Biogeography*. 00, 1-16. DOI: 10.1111/jbi.14986.

Saltré, F., Duputié, A., Gaucherel, C. & Chuine, I. (2014) How climate, migration ability and habitat fragmentation affect the projected future distribution of European beech. *Global Change Biology*. 21(2), 897-910. https://doi.org/10.1111/gcb.12771.

Thuiller, W., Georges, D., Guenuen, M., Engler, R., Breiner, F., Lafourcade, B. & Patin, R. (2023) *biomod2*: Ensemble platform for Species Distribution Modeling. R package version 4.2-4. https://cran.r-project.org/web/packages/biomod2/biomod2.pdf.

van der Linde, S., Suz, LM., Orme, CDL., Cox, F., Andreae, H., Asi, E., Atkinson, B., Benham, S., Carroll, C., Cools, N., De Vos, B., Dietrich, HP., Eichhorn, J., Gehrmann, J., Grebenc, T., Gweon, HS., Hansen, K., Jacob, F., Kristofel, F., Lech, P., Manninger, M., Martin, J., Meesenburg, H., Merila, P., Nicolas, M., Pavlenda, P., Rautio, P., Schaub, M., Schrock, HW., Seidling, W., Šramek, V., Thimonier, A., Thomsen, IM., Titeux, H., Vanguelova, E., Verstraeten, A., Vesterdal, L., Waldner, P., Wijk, S., Zhang, Y., Žlindra, D. & Bidartondo, MI. (2018) Environment and host as large-scale controls of ectomycorrhizal fungi. *Nature*. 558(7709), 243-248. <https://doi.org/10.1038/s41586-018-0189-9>.
